# Delocalization versus Coherence under Vibrational and Environmental Disorder in Photoexcited Supramolecular Aggregates

**DOI:** 10.1021/jacs.5c20341

**Published:** 2026-01-19

**Authors:** Samuele Giannini, Alekos Segalina, Daniele Padula, Marta Cantina, Mariachiara Pastore, Giacomo Prampolini, Fabrizio Santoro

**Affiliations:** a Institute of Chemistry of OrganoMetallic Compounds, National Research Council (ICCOM-CNR), Pisa I-56124, Italy; c Center for Advanced Reaction Dynamics, 364806 Institute for Basic Science (IBS), Daejeon 34141, Republic of Korea; d Department of Chemistry and KI for the BioCentury, Korea Advanced Institute of Science and Technology (KAIST), Daejeon 34141, Republic of Korea; e Dipartimento di Biotecnologie, Chimica e Farmacia, 9313 Università degli Studi di Siena, Via A. Moro 2, Siena 53100, Italy; f Université de Lorraine & CNRS, LPCT, UMR 7019, Nancy F-54000, France

## Abstract

Exciton and charge dynamics in photoexcited molecular materials depend critically on how delocalization competes with structural, vibrational, and environmental disorder. Yet, the origin and extent of coherent versus incoherent distribution of excitons and charges in such “noisy” supramolecular systems remain poorly understood. Here, we integrate all-atom classical dynamics with fully quantum vibronic dynamics to dissect these competing effects in aqueous self-assembled perylenediimide stacks. Our simulations quantitatively reproduce experimental absorption spectra and reveal that strong excitonic coupling and hybridization with charge-transfer states dictate the optical response. Following photoexcitation, the electronic populations rapidly spread across multiple molecules within tens of femtoseconds, yielding delocalized but largely incoherent exciton, hole, and electron wave function distributions. We find that high-frequency vibrational modes, and, to a lesser extent, the slow environmental and vibrational dynamics, intrinsic to the nature of such solvated supramolecular systems, set a fundamental limit to achieving fully coherent electronic delocalization. These results identify vibrational disorder as a universal constraint on coherent exciton dynamics and indicate that practical design principles for efficient organic optoelectronic and photocatalytic materials should focus on robust equi-distribution of the excited-state population.

## Graphical abstract

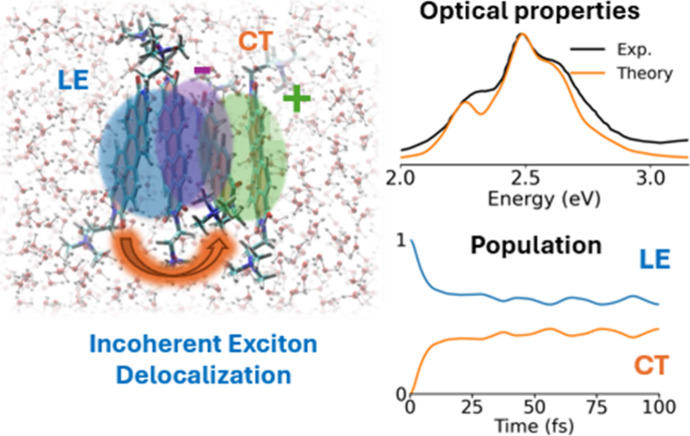

## 1 Introduction

Self-assembled π-conjugated molecular aggregates have attracted considerable attention due to their promising applications in photocatalysis and photovoltaic and optoelectronic devices.^1–3^ Among these systems, perylenediimide (PDI) self-assemblies have been extensively studied experimentally, owing to their structural robustness, tunable solvent-induced aggregation, high extinction coefficients, and strong π–π interactions.^1,3,4^ Thus, they represent a useful platform for bridging the gap between technologies based on single molecules, with important optical and photocatalytic properties,^5^ and those based on extended organic systems in which aggregation determines electronic and charge transport properties, promoting high exciton diffusion^6,7^ and charge carrier mobility.^8–10^

Electronic interactions among chromophores within molecular assemblies govern key processes such as excitation energy transfer, photoinduced electron (and hole) transfer, and intermolecular charge transfer, as well as the degree of delocalization of the exciton wave function.^11^ These processes are critical in optoelectronic applications, such as organic photovoltaics and photocatalysis, where efficient and directional transport of energy or charges to specific regions within the system is pivotal for driving electron transfer to a donor–acceptor interface^12^ or to activate a targeted catalyst.^13,14^ Additionally, other crucial factors influencing the photoinduced dynamics in these “soft” and often solvated materials include vibronic coupling, i.e., the interplay between electronic and nuclear vibrational motions,^15,16^ as well as the specific response of the environment (e.g., the solvent) upon excitation.^10^ In certain cases, these vibrationally and/or solvent-specific responses yield excimer formation,^17^ symmetry-breaking charge separation,^16^ and even lead to the singlet fission process.^4,16^ Moreover, the interaction between electronic and nuclear degrees of freedom can produce quantum phenomena such as vibronic coherences and delocalization, which have been proposed as mechanisms for enhancing function in both artificial and natural systems.^18–20^ For instance, ultrafast exciton delocalization has been detected in supramolecular neutral PDI assemblies.^21,22^ There remains, however, debate on the actual spatial extent of the exciton wave function and on whether such coherent delocalization can persist in the presence of the strong vibrational noise typical of molecular systems.^23^ A more fundamental understanding of the interplay between the actual atomistic structure, electron–vibrational dynamics, and related quantum phenomena would facilitate the development of design strategies to further improve the optoelectronic and catalytic performance of real devices. Providing a detailed understanding in this respect is the main objective of this work.

Computational studies have played a key role in unraveling the link between supramolecular organization and optical as well as transport properties in molecular aggregates. A widely adopted framework is Kasha’s model, which qualitatively relates spectral shifts to molecular arrangement: cofacial stacking typically results in a blueshifted absorption relative to the monomer (H-aggregates), while a head-to-tail alignment leads to a redshifted absorption (J-aggregates).^11,24,25^ These spectral signatures are closely related to the degree of exciton delocalization and coherence. For example, coherence is often inferred from emission spectra,^22,26^ but in low-emissive H-aggregates, this procedure becomes less reliable due to population transfer from the bright symmetric to the dark antisymmetric Frenkel exciton states, which quenches the 0–0 transition.

Additionally, several authors have shown that in closely π-stacked molecules, charge-transfer (CT) excitations, where the electron and hole occupy different chromophores, may interfere with local excitations (LE), significantly reshaping the absorption spectrum^27,28^ and/or modifying the transport of excitons.^29^ Nonetheless, the impact of CT states has been investigated mainly in terms of optical properties^27,30^ rather than with respect to exciton dynamics or wave function delocalization, leaving their role in enhancing or diminishing exciton coherence largely unexplored so far. Moreover, previous works have been primarily focussed on well-defined crystalline solid-state systems,^30,31^ which feature low structural (and therefore energetic) disorder, or idealized model systems, where the interaction between nuclear and electronic degrees of freedom is typically modeled through a single effective vibrational mode (e.g., a collective C=C stretching).^31^ In contrast, supramolecular aggregates exhibit much more disordered structures, and their electronic transitions can be coupled with multiple vibrational modes, especially when also CT states come into play. These complex systems therefore constitute a realistic testbed for investigating how both high- and low-frequency modes govern exciton delocalization and the enhancement or loss of coherence, calling for improved computational methodologies and a deeper theoretical understanding.

To address these aspects in solvated molecular aggregates, we employed a fully atomistic multiscale protocol that combines molecular dynamics (MD) sampling with full quantum vibronic dynamics, to investigate the excited-state properties of an aggregate of *N*,*N*-bis­(2-(trimethylammonium)­ethylene) perylene-3,4,9,10-tetracarboxylic acid diimide (PDI) formed in aqueous solution (Figure 1a).^32,33^ By explicitly accounting for structural disorder, solvent fluctuations, and their impact on electron–vibration coupling, our nonadiabatic simulations reproduce the experimental absorption spectra with remarkable accuracy and validate the structural and electronic Hamiltonian parameters, as discussed below. A distinct feature of our approach is the partitioning of the nuclear degrees of freedom into two sets: the slow ones, comprising the solvent and the intermonomer modes (and side chains), which are sampled classically, and the intramolecular ones, which are treated at the quantum-mechanical level, through nonadiabatic wavepacket (WP) propagation. This less explored picture of open quantum systems enables us to disentangle the role of fast vibrations from the slower environmental fluctuations, allowing an accurate probe, albeit on ultrafast time scales prior to thermalization and solvent relaxation, of their influence on key features of photoexcited systems, such as electronic delocalization and coherence. Our approach thus goes beyond weak-coupling perturbative treatments and Markov approximations, such as those used in the Redfield master equation.^34–37^ We show that while the wave function rapidly spreads across multiple sites, coherence is strongly damped within a few tens of femtoseconds owing to coupling with high-frequency vibrations. This result resolves a long-standing debate by demonstrating that in “noisy” molecular systems, where both low- and high-frequency vibrational and electrostatic environmental effects are present, exciton delocalization can survive, whereas coherence does not.

**Figure 1:**
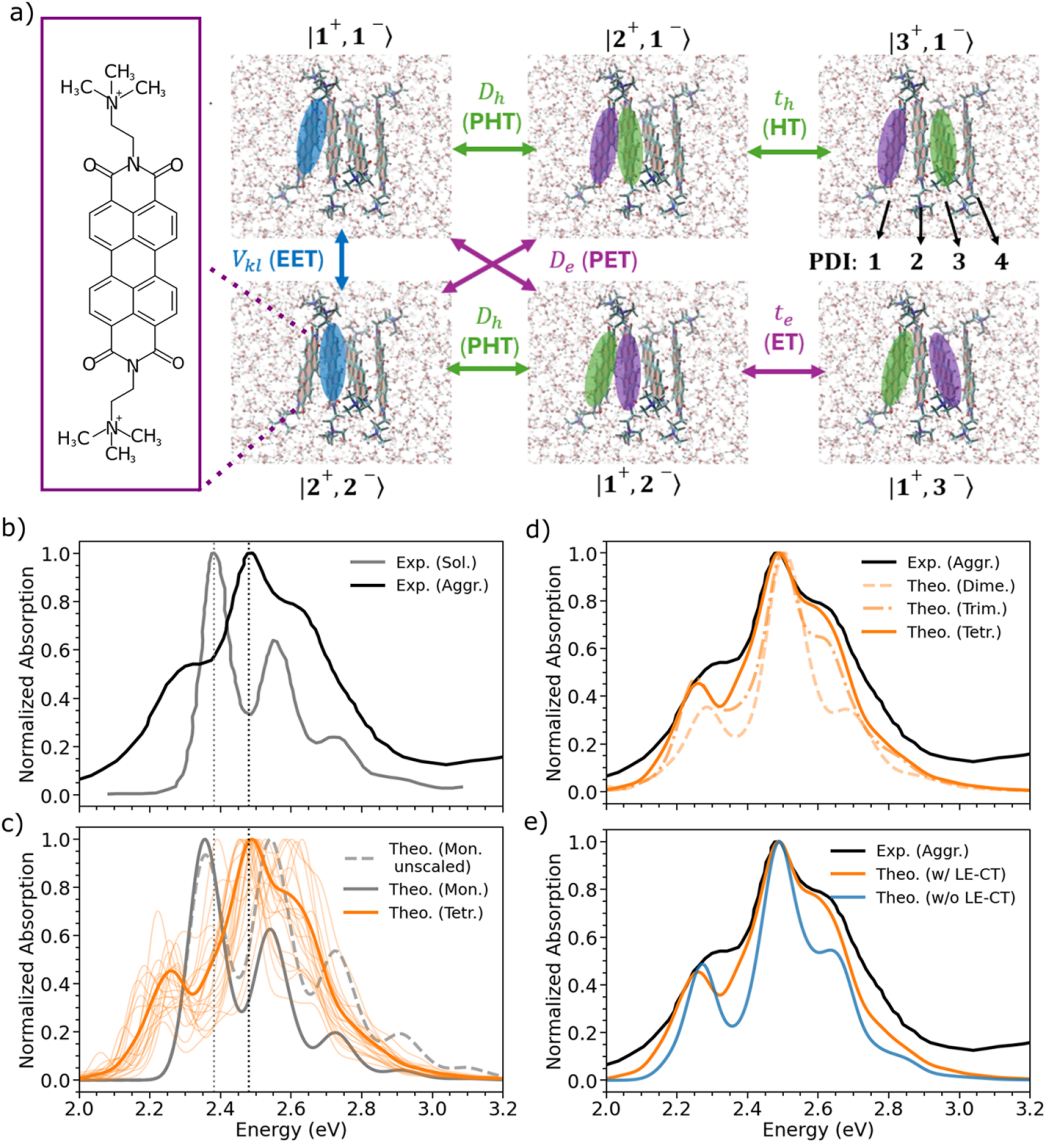
(a) Illustration of the PDI monomer (left) and tetramer (right) in explicit water solvent. Different electronic processes are illustrated. Exciton, hole, and electron particles are pictorially represented in blue, green, and magenta, respectively. Coupling between localized excitons (*V*_*kl*_) is shown in blue and leads to excitation energy transfer (EET). Photoinduced electron transfer (PET) and photoinduced hole transfer (PHT), responsible for exciton splitting and mediated by the electronic couplings *D*_*e*_ and *D*_*h*_, are shown in magenta and green, respectively. Electron and hole transfer processes, characterized by the transfer integrals *t*_*e*_ and *t*_*h*_, respectively, are indicated using the same color code to highlight the motion of the electron or the hole. Refer to Section S1.3 for further details. (b) Experimental absorption spectra^28^ for the monomer in dilute solution of acetonitrile (solid gray line) and the aggregate form (solid black line) in water. (c) Comparison of computed spectra for the monomer in vacuo and for a tetramer aggregate in water solution. The dashed gray line indicates the TDDFT spectrum obtained employing CAM-B3LYP energy gradients, while the solid line corresponds to spectra obtained by dividing such gradients by a factor of 1.3 (see Section S3.1). The calculated spectra were also shifted by 0.32 eV, and all transitions were convoluted with a Gaussian of HWHM = 0.05 eV for the monomer and 0.03 eV for the aggregate. Thin orange lines represent quantum dynamics (QD) trajectories calculated for each MD snapshot, while the thick orange line represents the averaged spectrum (eq S1). (d) Evolution of the aggregate spectrum as the system progresses from a dimer to a tetramer. (e) Comparison of the tetramer spectra including LE-CT couplings as in the other plots and removing them. Convergence with respect to the number of snapshots and convergence with the number of effective vibrations are given in Figures S10 and S11.

## 2 Methods

We refer to the mixed quantum-classical method employed here for the computation of vibronic absorption spectra of molecular aggregates and their nonadiabatic dynamics as Ad-MD|gLVC (which stands for adiabatic-molecular dynamics|generalized linear vibronic coupling model). This method addresses several key limitations of other simplified approaches present in the literature: (i) the lack of detailed structural information about the molecular assembly, (ii) the influence of the environment on the fluctuations of LE and CT excitation energies and their couplings, (iii) the need to incorporate vibronic coupling between several electronic states and multiple nuclear degrees of freedom, and (iv) the possibility of describing excited-state dynamics in a fully quantum-mechanical framework.

In brief, our approach, introduced in previous works^28,38^ and extended here to tackle larger and more complex supramolecular aggregates, relies on an adiabatic (Ad) separation between the soft low-frequency degrees of freedom (DoFs) and the stiff high-frequency vibrational modes. The soft and flexible coordinates, *R*, of the solute, and all the solvent coordinates, are treated at the classical level by sampling snapshots along MD trajectories carried out using accurate quantum-mechanically derived force-fields (QMD-FFs)^39,40^ that reliably describe the potential energy surface (PES) of the aggregate system. The stiff high-frequency modes, *r*, are treated instead using quantum dynamics (QD) of electron–nuclear WPs evolving on the coupled PESs described using linear vibronic coupling (LVC) Hamiltonians (eq S6). The electronic part of the Hamiltonian (eq S7) used to describe the aggregate is parametrized on the grounds of TDDFT calculations (CAM-B3LYP/6-31G­(d) level of theory), via an overlap-based diabatization performed with our freely distributed Overdia code.^41^ Such an approach, applied to aggregates of different sizes, allows us to retrieve excitation energies and electronic couplings between LE and CT states in the diabatic basis. The electrostatic effects coming from the surrounding water molecules are included through an ONIOM QM/MM approach. Details on the methodology such as the inclusion of the coupling to vibrations as well as the calculation of the Hamiltonian for specific solvent configurations are given in Section S1 in the Supporting Information.

The time evolution of the coupled electron–nuclear nonadiabatic dynamics is computed using the multilayer version of the multiconfiguration time-dependent Hartree method (ML-MCTDH)^42–46^ as implemented in the Quantics code.^39^ Fully converged low-resolution spectra, which account for the effect of virtually all nuclear coordinates, can be obtained by using a sufficiently large number of blocks of effective modes defined through a hierarchical representation of the LVC Hamiltonian (see details in Section S2.4). This strategy is defined in such a way that the short-time dynamics (the only one relevant for the low-resolution spectrum) is dominated by a few blocks.^47–51^ The soft DoFs are sampled via classical MD, carried out using accurate QMD-FFs.^39,40^ The coupling between slow and fast DoFs is incorporated by recomputing the key LVC Hamiltonian parameters for each uncorrelated MD configuration and then performing quantum dynamics (QD) for each of these snapshots, as explained below.

## 3 Results and Discussion

### 3.1 Experimental Observations and Structural Model

We begin our analysis discussing the experimental absorption spectrum of the PDI molecule in dilute acetonitrile solution (where PDI is completely soluble) shown in Figure 1b (gray curve). The well-resolved vibronic progression commonly seen in many π-conjugated molecules is clearly visible and has an origin (0–0, gray dotted line) at approximately 2.38 eV. By using TDDFT calculations in combination with a (quantum) displaced harmonic oscillator model^33,52^ on the optimized single molecule structure in vacuo, we could reproduce the main spectral features observed in the experiment (Figure 1c, details in Section S3.1). We note that a detailed and accurate description of the monomer in solution, including solvent effects, was already provided and thoroughly validated in our previous study.^33^

Analysis of the vibronic progression reveals that several modes contribute to the fine structure of the spectrum. High-frequency stretching modes around 1360–1690 cm^–1^ are responsible for the main vibronic progression, while more delocalized low-frequency modes (e.g., those around 234 cm^–1^) are involved in the broadening of the peaks as previously noted.^33^ A second point is that our chosen functional, CAM-B3LYP, tends to overestimate the vibronic coupling, that is, the 0–1 transition is too intense compared to experiment (see Figure 1c).^33^ This overestimation is corrected in this work by dividing the vibronic gradients by a factor of 1.3 as explained in Section S3.1.

Analysis of the aggregate spectrum of PDI in aqueous solution reveals pronounced spectral changes and a redistribution of oscillator strength among the main peaks, as shown in Figure 1b. For example, the intensity of the 0–0 peak decreases, while the 0–1 peak becomes the most prominent compared to the monomeric spectrum. Aggregation also induces a substantial spectral broadening and a blueshift of approximately 0.1 eV in the main band of the aggregate relative to the 0–0 maximum of the monomer in solution. These changes highlight the critical roles of excitonic effects, energetic disorder, and vibronic interactions, whose individual contributions are analyzed in the following. A critical factor to consider when modeling the optical changes induced by aggregation is the size and arrangement of the molecular aggregates formed as the PDI concentration increases. We addressed this challenge using advanced sampling MD simulations,^53^ supported by accurate QMD-FFs. These simulations revealed that PDI molecules in solution tend to aggregate anticooperatively, by predominantly forming and growing through dimer-based associations driven by entropic contributions arising from the release of solvating water molecules.^53^ Notably, employing enhanced sampling techniques, we found that, although aggregates of various sizes can be formed, they represent only a minor fraction of the population, with tetramers representing the predominant species (see Figure 1a). These simulations also allowed us to characterize the relative motion of the monomers within the aggregates, which exhibit substantial structural heterogeneity and internal disorder, as discussed below.

### 3.2 Electronic Hamiltonian Parameters

To investigate the influence of structural disorder on the electronic parameters, we built upon the previously identified aggregate structures, to construct electronic Hamiltonians for aggregates of different sizes (dimers, trimers, and tetramers) on the basis of diabatic LE and CT states, using the diabatization strategy implemented in our Overdia code.^41,55^ We constructed LVC Hamiltonians (eq S6) of the aggregates using snapshots extracted every 10 ns from a 500 ns MD trajectory,^53^ thereby capturing both structural and energetic disorder. For each MD snapshot, as detailed in Section S2.2, we performed a DFT-constrained geometry optimization of the PDI core of each individual monomer within the aggregate, while keeping the lateral chains and neighboring PDI units fixed. This procedure allows us to disentangle the effect of the fast high-frequency modes, already treated separately in the vibronic part of the Hamiltonian, and the dynamics of the slow modes, included at the classical level through the MD sampling. For illustrative purposes, we depict in Figure 2a the average over ∼45 snapshots of the Hamiltonian describing the tetramer system.

**Figure 2:**
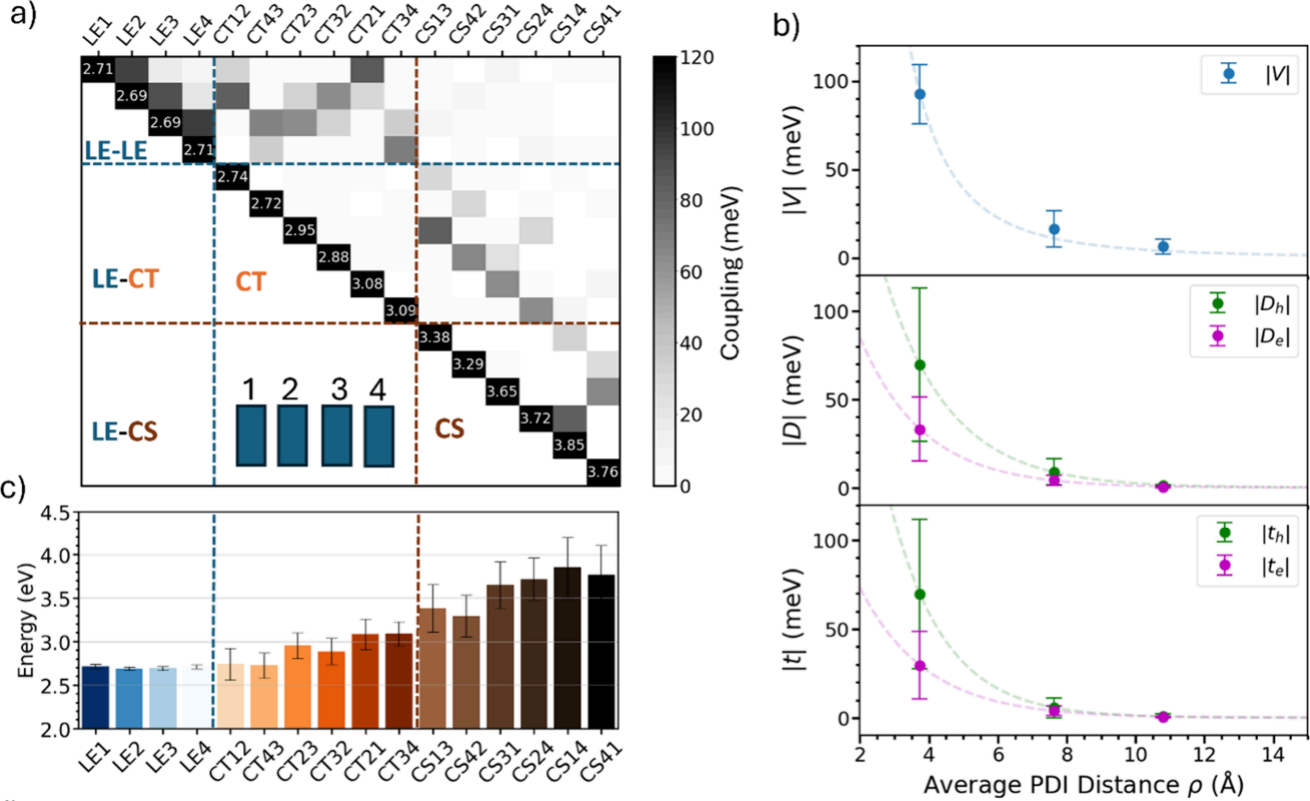
(a) Average Hamiltonian matrix elements and coupling blocks: LE blocks containing the excitonic interactions between LE states, LE-CT and LE-CS blocks containing interactions between LE and CT states and LE and charge-separated (CS) states, and CT and CS blocks containing the interaction between electrons and holes sitting on different molecules. The inset shows the order of the molecular sites over which the states are localized. Excitation energies reported on the diagonal are given in eV. With CT*_kl_*, we indicate the state in which the electron has moved from molecule *k* (first index) to molecule *l* (second index). See also Figure 1a for a visual representation of the interactions. (b) Excitonic couplings (*V*), photoinduced charge-transfer couplings (*D*), and transfer integrals (*t*) as a function of distance and their fluctuations. Note that couplings are shown in absolute values for clarity, but a consistent coupling sign relationship between all states is fundamental to properly describe the optical spectra and the excited state dynamics (see discussion in refs 30 and 56 ). Our diabatization takes this correct relation into account. (c) Excitation energies ordered to show the Coulomb-like trend as a function of distance.

As expected from the close cofacial stacking distance with an average value of 3.7 Å, the aggregate exhibits large H-type excitonic interactions (*V*), reaching up to ∼94 meV on average (see Figure 2b). We also observe sizable photoinduced hole transfer couplings (PHT), *D*_*h*_, reaching up to ∼72 meV. PHT couplings connect a local excitation LE state, |e_*k*_⟩, of a monomer *k*, to a nearby CT state, |c_*k*+*s*_, a*_k_* ⟩, a*_k_* being the anion with the electron on monomer *k* and c_*k*+*s*_ the cation with the hole located on monomer *k*+*s,* where *s* is the distance to another site that the hole has traveled to (see Figure 1a and eq S10). Despite averaging over multiple snapshots, PHT couplings remain significantly stronger than the corresponding photoinduced electron transfer (PET) interactions, *D*_*e*_ ∼33 meV, which represent the coupling between the exciton on monomer *k* and the CT obtained by transferring an electron to *k*+*s*, thus indicating that holes are expected to be more mobile than electrons. A similar trend is observed for the ground-state hole and electron transfer integrals (*t*_*h*_ and *t*_*e*_, respectively), which describe charge transport between CT states (see Figure 1a and eq S9). Interestingly, the photoinduced couplings and related transfer integrals, which are primarily governed by orbital overlap, are strongly influenced by disorder, i.e., the low-frequency intermolecular structural fluctuations, as witnessed by their large standard deviation, σ (see data in Figure 2b). For example, the σ of *D*_*h*_ and *D*_*e*_ accounts for approximately 60% of the total coupling values for the closest nearest-neighbor interactions (see Table S4), while it is reduced to 16% for excitonic *V* couplings, confirming that the latter is relatively robust against structural disorder.

Besides electronic couplings, another fundamental ingredient is the excitation energy of the constructed diabatic states as represented in Figure 2c. LE excitation energies exhibit relatively small fluctuations (∼20–26 meV) across different molecular sites, indicating weak sensitivity to structural and environmental disorder (i.e., the energetic disorder induced by interactions with surrounding water molecules). Interestingly, LE2 and LE3 excitations in the central region of the tetramer show a slightly smaller energy compared to LE1 and LE4 due to polarization induced by the external PDI units. As expected, CT energies are significantly more sensitive to the slow configurational dynamics of the assembly and the polar aqueous environment, showing larger fluctuations across different snapshots (∼150–180 meV). Notably, this effect becomes more pronounced for CT states involving an electron and a hole located on nonadjacent molecules. This is the case for long-range CT states (referred to as charge-separated states, CS) at the extremes of the aggregate (i.e., CS14 and CS41), which show the largest energy fluctuations (340–345 meV). As expected, when the electron and hole are located farther apart, the energy of the CT states increases due to a Coulomb-like barrier. Long-range CT states, therefore, lie significantly higher in energy compared to nearest-neighbor CT states, hinting at the inherent difficulty of charge separation. As we will discuss below, these CS states are not easily populated during the initial photoexcitation dynamics.

To gain a deeper understanding of the origin of the observed fluctuations and their connection with the sampled molecular geometries, we quantified how structural motions modulate the electronic Hamiltonian (see Section S3.4). To this aim, we correlated geometric descriptors from each MD snapshot with the corresponding Hamiltonian elements. The results in Figure S8 report the Pearson and Spearman correlation coefficients, capturing linear geometry–coupling relationships. Distance-type descriptors, such as the center-of-mass separation (ϱ) and the aromatic-plane separation (*r*_π_), exhibit the strongest correlations with the off-diagonal Hamiltonian terms and with the CT excitation energies. In contrast, angular descriptors are generally less influential; among them, the yaw rotation (α) plays a primary role, reflecting collective tilting motions of the PDI stack that modulate cofacial alignment and excitonic coupling. A secondary yet non-negligible contribution arises from the dephasing angle (δ), which more effectively captures concerted columnar distortions and collective reorganization along the aggregate axis.

The importance of considering explicitly electrostatic solvent effects on electronic parameters was tested by performing diabatization in vacuo on a reduced set of snapshots used to construct the Hamiltonian in Figure 2. Our simulations (see Figure S9) reveal that explicitly accounting for the solvent and its electrostatic and polarization effects has only a minor influence on the LE energies and coupling fluctuations, but a pronounced effect on the energies of CT states, consistent with the expectation of strong local interactions settled with specific solvent molecules. In particular, the asymmetry between CT12 (CT43) and CT21 (CT34) energies is enhanced in the gas phase compared to water, suggesting a preferred pathway for the electron to localize toward the center of the oligomer stack. Water molecules tend to screen the strong positive charge generated by the ammonium ions of the alkyl chains, thereby reducing the driving force for the electron to move toward the middle of the system. These effects strongly influence the hybridization of LE and CT states and the resulting excited-state dynamics as discussed below. Similar fluctuations in the electronic parameters were also observed for dimers and trimers (see Figure S6), with the notable difference that excitonic interactions between LE and CT states in smaller aggregates lead to a distinct eigenstate spectrum and modified spectral intensities of the vibronic bands, as we discuss in the following sections.

### 3.3 Simulated Optical Properties

In Figure 1c, our simulations, when averaged over several QD spectra obtained from aggregate snapshots characterized by different supramolecular tetramer structures (thin lines), show remarkable agreement with the experimental data, both in the blueshift observed upon aggregation and in relative peak intensities (see the thick line). This agreement is achieved without introducing any adjustable phenomenological parameters in our model, apart from a global spectral shift applied to correct the absolute energies due to DFT inaccuracies.^33^ The vis-à-vis comparison with experiments indirectly validates our previous structural simulations, which predicted tetramers to be the predominant species.^53^ Our simulations in Figure 1d show that the spectrum significantly changes going from a dimer to a trimer, whereas only minor differences are observed when going from the trimer to the tetramer, with tetramers showing the best agreement with experimental data (see the comparison between Figures 1b and 1c). The spectral changes observed upon aggregation, from smaller to larger aggregates, are also fully consistent with experimental studies on similar PDI dimer and aggregate systems.^22,54^

Achieving quantitative agreement with experiments is only possible when the full atomistic details of the structure and its fluctuations are considered. Interestingly, as shown in Figure S12, using only an averaged Hamiltonian (i.e., a Hamiltonian constructed with parameters averaged over all the snapshots) results in noticeable discrepancies of the simulated spectrum with respect to experiments. This finding highlights that neglecting fluctuations, or treating them in an averaged manner, is not sufficiently accurate. Our simulations reveal an intimate relationship between the electronic structure and specific (supra-)­molecular morphology. This is further evidenced by the distributions of coupling strengths shown in Figure S7, which reveal skewed, non-Gaussian fluctuations, whose instantaneous values depend on the specific molecular arrangement. This observation indicates that applying uncorrelated Gaussian noise to the parameters to mimic disorder in a solid-state environment, as it is common practice in the literature,^11,56^ would result in a loss of accuracy for solvated supramolecular aggregates.

In Figure 1e, we show that another important factor influencing the spectral shape is the hybridization between LE and CT states. When couplings between LE and CT are neglected (blue line), the spectrum changes dramatically both in terms of peak position and overall broadening, resulting in a much poorer agreement with experiments. As represented in more detail in Figure S13, unlike in the dimer, where a relatively weak hybridization between LE and CT was observed,^28^ in the tetramer, the LE/CT mixing is largely enhanced by the increased density of CT states. Such a mixing causes redistribution of the oscillator strength among bright LE states and initially dark CT states, which borrow intensity from the former, thereby changing the spectral shape and leading to a better match with the experiments. As we discuss in the following, a similar effect is also clearly visible in the population of the excited states.

### 3.4 Ultrafast Excited-State Nonadiabatic Quantum Dynamics

QD simulations give us a deeper insight on the excited-state dynamics at short time scales and the evolution of electronic populations across the PDI aggregate’s units in the condensed phase.^57^ In particular, Figure 3a shows that immediately after photoexcitation of the local exciton state of the first monomer (number 1, LE1), a rapid population transfer occurs. Within 10 fs, part of the energy is transferred to the second monomer, and after an additional 10–15 fs, the third and fourth monomers also acquire a considerable population. In 100 fs, the central LE2 and LE3 states become slightly more populated as they are slightly lower in energy than LE1 and LE4. Interestingly, during the energy transfer process, there is a simultaneous rise in the CT21 population, triggered by a fast photoinduced hole transfer from LE1, facilitated by the strong PHT coupling (*D*_*h*_). Nevertheless, since this state lies ∼350 meV higher in energy than the corresponding CT12, it becomes depopulated within 20 fs. CT12’s population, on the other hand, begins to grow due to rapid PHT from LE2 and, possibly, a slower PET process from LE1. The analogous CT43 state also becomes populated by 15%, albeit with a slower rise following the population of LE3. The impact of LE/CT mixing is evident in Figure 3b, which shows the summed populations of states of the same nature. Consistent with optical spectra of aggregates of different lengths, our QD simulations reveal that CT states are rapidly and substantially populated, reaching about 40% in the tetramer compared to 30% in the dimer. This finding underscores the crucial role played by the higher density of CT states.

**Figure 3:**
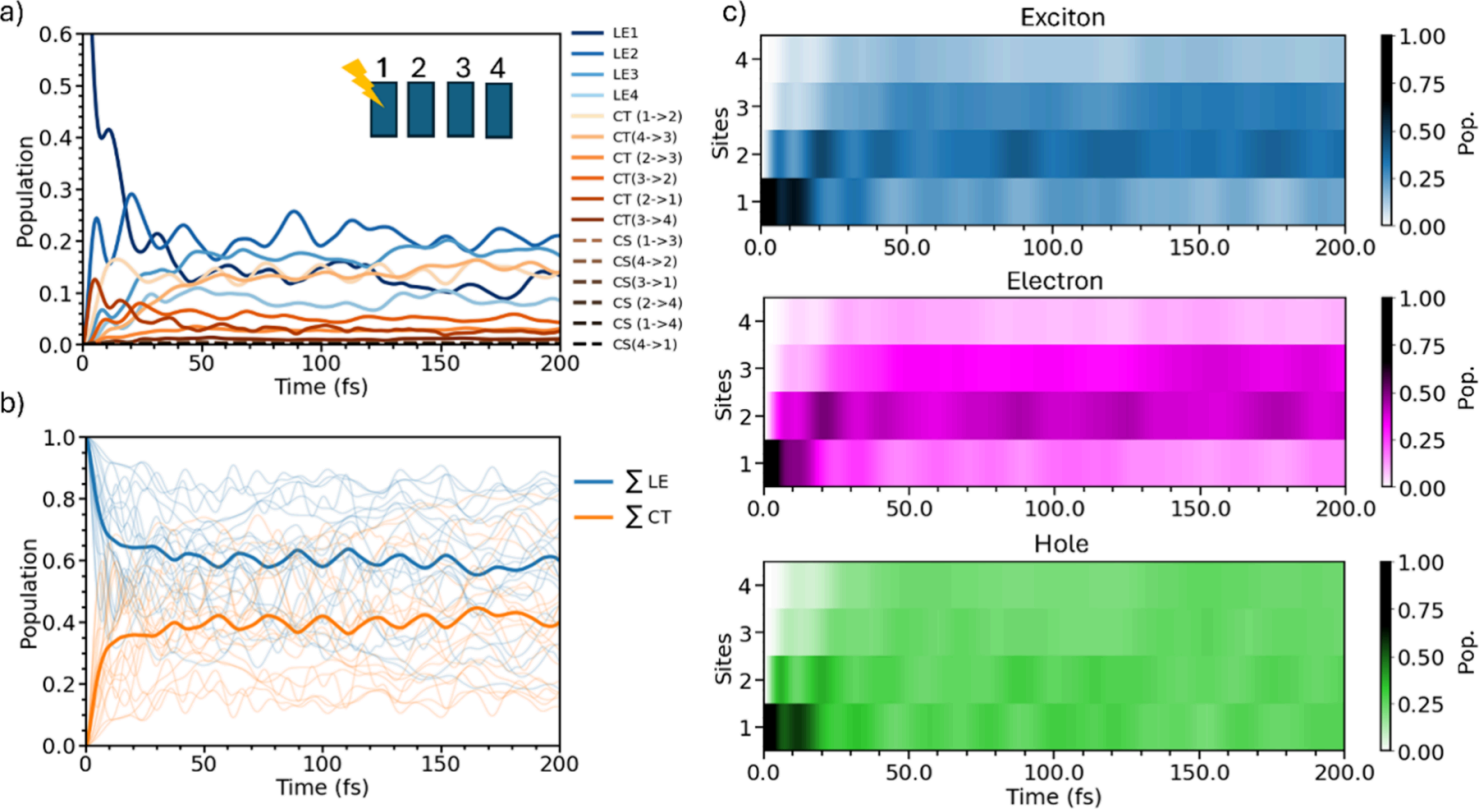
(a) Diabatic population as a function of time. The population is averaged over several quantum trajectories with a different electronic Hamiltonian. (b) Evolution of the summed population of LE states and CT states for individual trajectories (thin lines) and averaged over all trajectories (thick lines). (c) Population of LE, electrons, and holes for the different sites as a function of time. Note that the hole (electron) population is obtained by considering the corresponding electron (hole) as being anywhere (see equations in Section S1.7).

A final observation is that CS states, being high in energy, do not become significantly populated. In the limit of our model, therefore, exciton separation beyond two neighboring monomers appears energetically unfavorable, although the situation may change when accounting for the possible dynamical response of the solvent at longer times (which may stabilize the large dipole of CS states) or with the inclusion of an electric field to help separating charges. As a matter of fact, the solvent reorientation might stabilize CS states, thereby enhancing their population and facilitating exciton splitting. This picture is fully consistent with experimental observations made in ref 16, where a symmetry-breaking charge separation effect, induced by the solvent relaxation and low-frequency interchromophore vibrational modes, was reported for a PDI trimer derivative in a longer 2–3 ps time scale. In a similar way, an electric field in the direction of the chain would lower the Coulomb barrier between electrons and holes at longer distances, hence stabilizing CS states.^58^ Before proceeding further, we also note that, in principle, triplet excitons, although not directly optically accessible, may also form in PDI aggregates, for instance via singlet fission. This is because correlated triplet pairs can mix with LE states or be populated via CT states. Such processes generally occur on longer time scales than those explored in this work, although they have been observed in PDI aggregates depending on their morphology and molecular arrangement.^59,60^ The methodology presented here could be adapted to include higher spin states (e.g., triplets) by extending the basis set of the electronic Hamiltonian. In such a case, a proper benchmarking of the coupling interactions with singlet states and of the energies of localized triplet states would be required.

As mentioned in the Methods section, QD calculations are performed at 0 K for a static representation of the slow degrees of freedom. This is expected to be a good approximation on the ultrafast time scale since only the fast modes (>1400 cm^–1^, corresponding to energies larger than ∼7 *k*_B_ *T*) are active on this time scale. We also note that QD is more conveniently performed in the local diabatic representation, rather than in the adiabatic one, where nonadiabatic couplings between potential energy surfaces become spiky at the crossing points.^61^ Nevertheless, to provide insight into the time evolution of delocalized states closer to the adiabatic ones, we can use the adiabatic-to-diabatic transformation at the reference Franck–Condon point to project the WP, as shown in Figure S14b. These new delocalized states, identical to the adiabatic ones at the FC point, are simply labeled as S1–S16. Our simulations clearly indicate that the initial excited WP relaxes toward the lowest excited S1 state at the bottom of the exciton band, which hosts about 50% of the total population at 200 fs. Further relaxation could occur on longer time scales, when slow modes, treated as static in our simulations and therefore acting as “spectator modes”, begin to relax and exchange energy with the fast modes after photoexcitation. These slow modes, which are included only through classical statistical sampling prior to excitation, may facilitate the dissipation of excess kinetic energy and promote further wave function relaxation. This effect, as well as the fulfillment of the Boltzmann equilibrium distribution for the whole system, could in principle be achieved by switching to a semiclassical approximation for solving the electron–nuclear equations of motion.^62^

Beyond the time evolution, several studies have shown that the ability of the exciton population to delocalize over multiple sites plays a key role in enhancing functions such as electronic transport and charge separation in molecular solids and heterojunction interfaces.^7,63–65^ To understand such a spatial evolution, in Figure 3c, we defined (renormalized) populations of the exciton, hole, and electron on the basis of the (diabatic) molecular sites according to eqs S20–S22. From this plot, we clearly see that the exciton and the electron preferentially travel toward the center of the aggregate on an ultrafast time scale, following the energy distribution displayed in Figure 2c. In contrast, the hole population spreads across the different sites. From an application perspective, being able to identify the sites to which electrons and holes migrate is crucial. For instance, it could enable docking of a catalyst precisely at the location where the electron population accumulates, hence facilitating its catalytic function.^5^

As we discuss below, several metrics can be used to quantify the extent to which excitons, holes, and electrons delocalize/distribute across different sites. Such a delocalization of the wave function depends on the interplay among many electronic parameters, including the magnitude of excitonic interactions, their coupling to vibrational modes, their fluctuations, and the degree of energetic disorder present in the system. Interestingly, Figure 3c shows that while the wave function spreads, its population exhibits quantum beats, most evident during the first 40 fs, due to coupling with the fast vibrational motion. These beats, which suggest somewhat coherent (synchronous) WP motion, are particularly evident in the diabatic state population shown in Figure 3a: when the population of the LE1 state reaches a maximum, that of the LE2 state is at a minimum, and vice versa. A similar behavior is observed for CT12, while the populations of the CT21 state exhibit weaker oscillations induced by vibrational coupling.

Taken together, these observations demonstrate that the system displays rich ultrafast photophysics and excited-state dynamics, characterized by coupling between electronic and nuclear degrees of freedom (vibrational coherences) that persist, at least transiently, despite the energetic disorder introduced by very slow (to be considered almost static) environmental heterogeneity. These observations give rise to two important questions: Is the delocalization in the electronic population truly coherent? Also, to what extent do the coupled electron–nuclear dynamics sustain coherent delocalization of excitons, holes, and electrons, despite the noisy nature of the environment?

### 3.5 Quantum Delocalization and Coherence

A widely used measure to quantify delocalization on different molecular sites of excitons, holes, and electrons based on their populations is the inverse participation ratio (IPR), defined in eq S23 as an average over the QD time evolution of populations on the individual snapshots. The IPR is a basis set-dependent quantity, which, in the basis of the molecular sites, correlates with the number of molecules (i.e., PDI monomers) *N* over which the electronic population is delocalized. For a fully localized particle, the IPR is 1, while it sums up to *N* (in our case 4) for an equally distributed mixture. The same limiting values can be obtained for electrons and holes using the generalized definition reported in Section S1.8. In Figure 4a, we show the IPR time evolution after excitation to LE1, which grows and then saturates in 40 fs to ∼3.3–3.4 for both holes and excitons due to the large excitonic interactions (*V* and *D*_*h*_, respectively) and the small electron–vibrational coupling (see Tables S1–S3). Because of the smaller *D*_*e*_ and its larger relaxation energy, the behavior registered for the electron is different: the IPR converges to a lower asymptotic value (∼2.8), pointing to a less delocalized wave function with respect to the one obtained for the hole and not completely spread over the whole aggregate. As discussed by Scholes et al.,^66,67^ it is important to note that the IPR does not distinguish between the two opposite scenarios of a completely incoherent distribution of localized electronic states and a fully delocalized electronic coherent state. Such a fully delocalized and coherent state would correspond to a vibronic state characterized by identical vibrational wavepackets (in both the coordinate and momentum space) traveling on all states with equal populations.^68^

**Figure 4:**
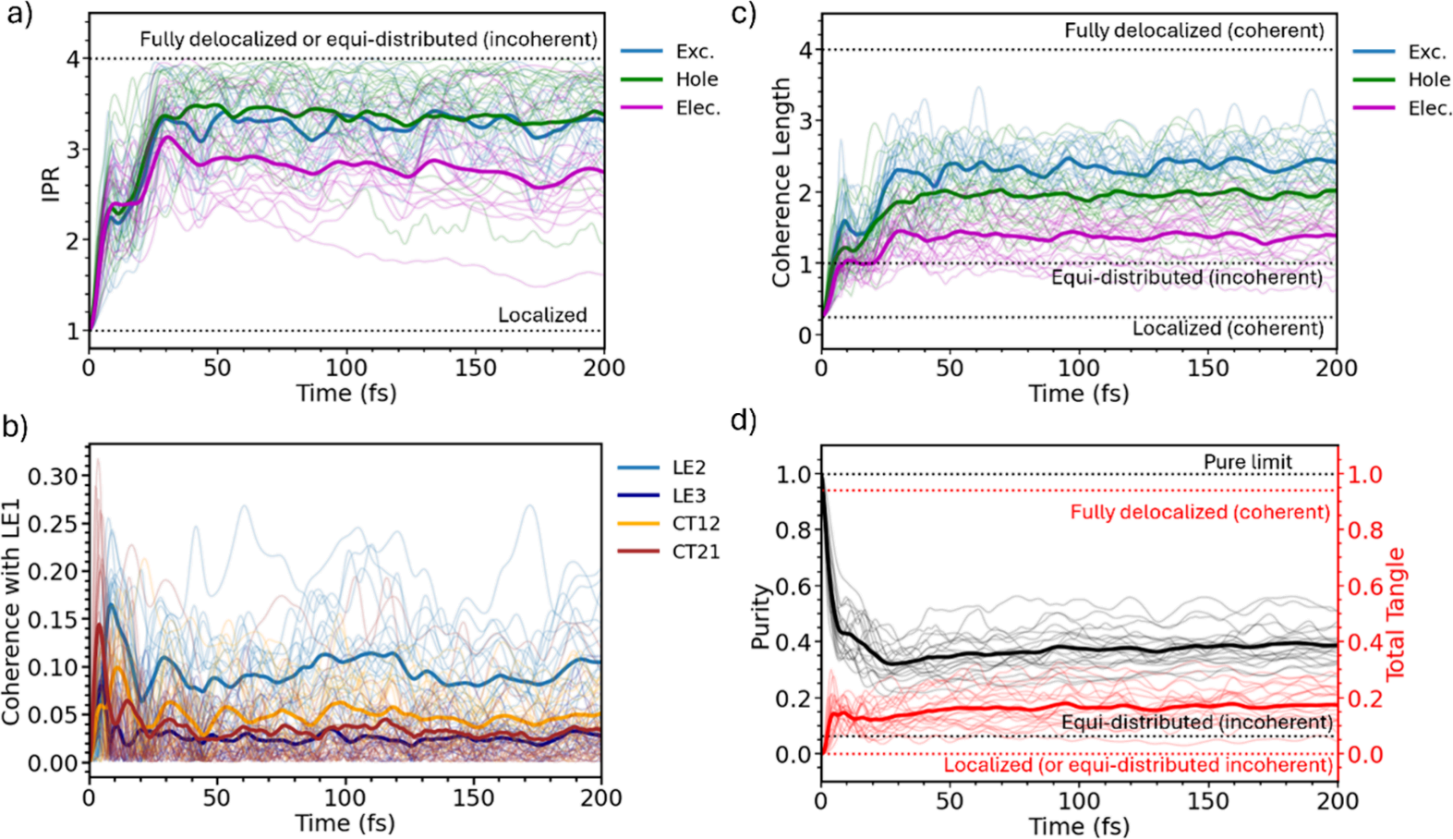
(a) Inverse participation ratio (IPR) of the exciton, hole, and electron (blue, green, and magenta, respectively) computed with eqs S23–S25. (b) Real part of the coherence between the LE1 state and the indicated diabatic states (the absolute value of the coherence follow a similar trend, see Figure S18). (c) Coherence length for excitons, holes, and electrons (blue, green, and magenta, respectively) computed with eqs S23–S25. (d) Purity of the electronic density matrix, defined in eq S18 (main axis). Total tangle, defined in eq S27 (second axis). In all plots, thin lines represent the quantity computed for each trajectory initiated from a different MD snapshot, while thick lines represent the average over all trajectories. Dashed lines indicate the theoretical limits, as discussed in the main text and Section S1.

To shed further light on this aspect, it is necessary to undergo a more in-depth analysis of the density matrix (ρ) of the system (eq S15). Our analysis has focused so far only on the electronic populations, i.e., the diagonal elements of the electronic reduced density matrix ρ_el_(obtained by tracing on the vibrational states, see details in Section S1.5). We now examine the off-diagonal elements of ρ_el_ that represent electronic coherences, focusing on those between the initially excited LE1 state and the ones to which this is most strongly coupled. The evident initial growth of the computed values displayed in Figure 4b points to a fully coherent transfer of population. This occurs because the initial vibrational WPs on the different electronic states remain similar in both the coordinate and momentum space.^68^ However, after a dozen femtoseconds, all these coherences saturate to smaller, although nonvanishing, values.

An additional measure of electronic delocalization based on reduced density matrices, known as coherence length (CL), was introduced in ref 69 and used by other authors^67,70^ to quantify the coherent delocalization of a wave function. We should mention here that to compute this quantity for electrons/holes, we defined new reduced electronic density matrices where the trace was performed not only along the vibrational states but also on the hole/electron states, see eq S26. The CL is 1/*N* for a fully localized state, 1 for an equally distributed (equal populations) and totally incoherent mixture of states (zero coherences), and *N* for a fully coherent and equally distributed state. Figure 4c shows that, after starting from a fully localized (and pure) state, the CL for all particles increases above 1 in less than 20 fs and reaches larger values for holes and excitons than for electrons, similar to what we observe for the IPR, remaining in all cases well below 4. This finding confirms that the state tends to distribute over all sites, but not in a fully coherent way, and that localization and possibly the loss of coherence are larger for electrons (which exhibit stronger electron–vibrational coupling, as reported in Tables S1–S3).

A synthetic description of the purity of the electronic state (i.e., the possibility to be described by a single quantum state vector) is given by the trace of the squared electronic density matrix, Tr­(ρ_el_^2^), see Section S1.5. This quantity has known limiting values of 1 for a pure (coherent) state and 1/*N* for a fully incoherent mixture of states. In line with the observation made for IPR and CL, we show in Figure 4d that this quantity exhibits a rapid drop from 1 to a value of 0.4, indicating both a rapid increase in the mixing of the states and an incomplete but substantial loss of coherence. A closely related quantity is the von Neumann (VN) entropy discussed in Figure S16. Although the purity and VN entropy are very interesting basis set-independent measures of mixing between states, they are not a measure of coherent delocalization over the different sites of the molecular aggregate. Moreover, they are computed considering the totality of the excited states, irrespective of their LE or CT character.

According to Scholes et al.,^67^ a more balanced estimate of coherent delocalization over *N* molecular sites can be retrieved from the relative entropy (eq. S29) and the total tangle (eq. S27). These quantities are 0 for both a state localized on one site and an incoherent state equally distributed on all sites, whereas they reach 1–1/*N* for a fully coherent state, equally delocalized on all sites. We focus on the total tangle (as defined in Section S1.10) and reporte this quantity on the right *y*-axis of Figure 4d. Considering all states, the tangle is initially null (the excitation is localized on site 1, LE1) and then saturates very quickly to approximately 0.15, far from the value expected for a fully delocalized and coherent state (0.94). This trend partially mirrors the one observed for the purity. We note that the total tangle for holes and electrons respectively saturate at 0.1 and 0.04 (Figure S17e), indicating that they are also far from being coherent across the different sites since the limit value for a fully delocalized coherent state is 0.75. The smaller value for electrons is likely due to their stronger localization on the middle molecules 2 and 3 as observed also in the population dynamics. As a further interesting note, we found that, although CT states have a clear impact on the spectral and dynamical properties of this system, their existence has a minor impact on exciton delocalization (see Figure S18), in line with what was found by some of us for a simplified model,^29^ in the presence of H-aggregates with small positive LE-CT offsets.

Overall, all measures of electronic delocalization within the molecular aggregate consistently indicate that, within just a few femtoseconds after photoexcitation, the WP in the PDI tetramer becomes distributed over at least three sites, in very good agreement with previous spectroscopic estimates on similar PDI aggregates.^17,22^ Importantly, we found that this holds true regardless of whether the initial state is prepared as a fully localized diabatic state or as a delocalized bright state, similar to the one that would be experimentally prepared by a broadband laser pulse (see discussion in Section S3.13). At the same time, our analysis of the density matrix reveals that, at least for the supramolecular assemblies investigated here, although there is a distribution of the excited population on different sites, such a delocalization is rather incoherent: the WP distributes on different monomers, yet its components are moving in different directions both in the coordinate and momentum space, thereby significantly reducing their overlap over time and space. The microscopic origin of such a coherence loss is investigated in the following.

### 3.6 Vibrational Dynamics and Coherence Loss

A striking outcome of our analysis in Figure 4 is that the loss of purity is governed by the coupling between electronic states and fast vibrational modes, rather than by the disorder introduced by the aggregate’s and solvent’s slow dynamics. The latter introduces only moderate variations across different snapshots (thin black lines in Figure 4d). Thus, the mixing between states and the associated loss of coherence originate mainly from dephasing effects induced by the motion of quantum high-frequency degrees of freedom. On the one hand, these vibrations are essential for promoting delocalization by spreading the wave function over multiple sites; on the other hand, they simultaneously reduce coherence, i.e., the overlap between WPs evolving on different electronic states.

This dual role is illustrated in Figure 5, where we report the purity and the total tangle as defined in eq. S27 (averaged over all snapshots) for three different scenarios: (i) the physical system discussed so far (with vibronic gradients reduced by a factor of 1.3), in which the coupling to high-frequency vibrations yields very good agreement with the experimental optical spectra, (ii) a model system in which this coupling is reduced by a factor of 10, and (iii) an ideal model where the coupling to high-frequency vibrations is completely removed. After a localized excitation, reducing the electron–vibrational coupling by an order of magnitude results in a more coherent delocalization (total tangle ≈ 0.35 vs 0.15 in the physical system). In the extreme limit where such couplings carried out by fast mode are neglected, the electronic state remains, by definition, pure, provided that low-frequency modes are assumed to be so slow to be considered frozen. It is, however, important to highlight that even in this extreme case, disorder arising from low-frequency molecular and solvent fluctuations, prevents full coherent delocalization: the tangle saturates at 0.75 instead of the ideal 0.94. Thus, beyond the pronounced impact of high-frequency modes, this residual slow dynamics contributes to a disordered initial Hamiltonian of the supramolecular aggregate, already present at photoexcitation step, eventually limiting the degree of delocalization among sites. Even at the purely electronic level, the instantaneous Hamiltonians corresponding to different slow configurations are not perfectly symmetric, leading to intrinsic Rabi-like oscillations at different times in the electronic populations that are effectively damped upon ensemble averaging. We note in passing again that these trends in vibrational dynamics and coherence loss generally hold true even when the system is prepared from a bright adiabatic state (see Section S3.13).

**Figure 5:**
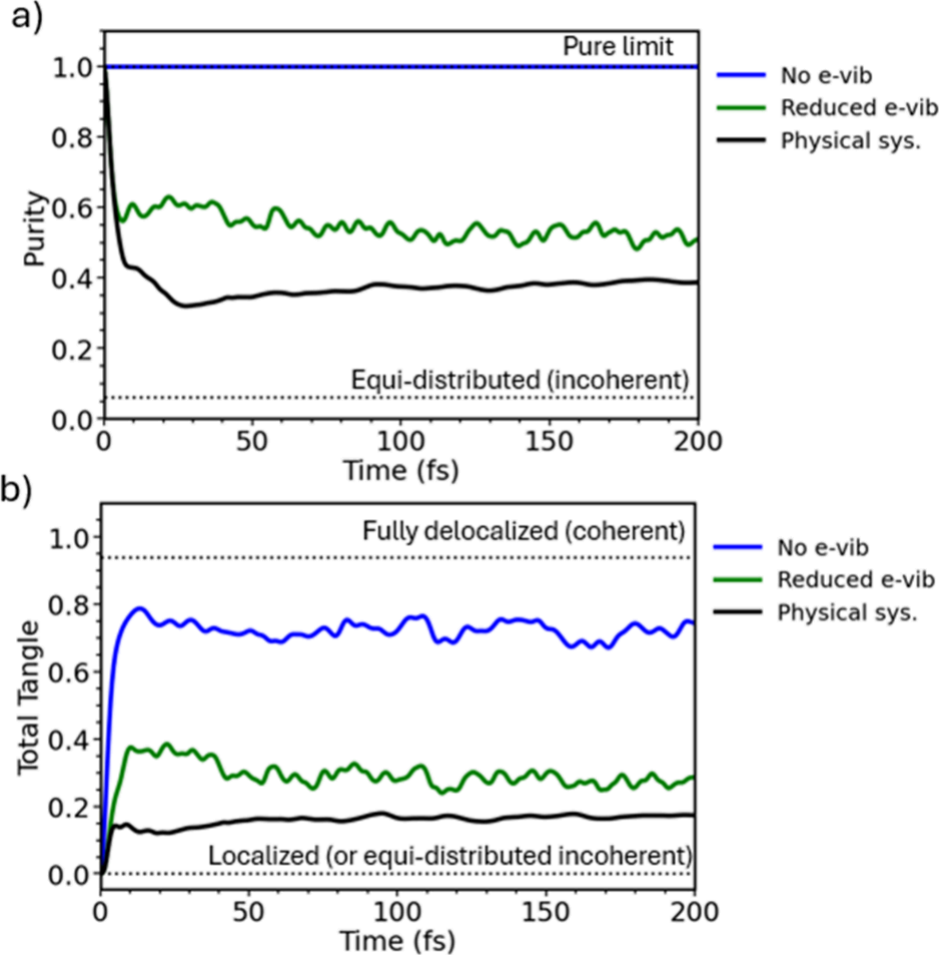
(a) Purity of the electronic density matrix, defined in eq S18, and (b) total tangle, defined in eq S27. Both these measures are performed for the physical system (with scaled gradients), in which the coupling to high-frequency vibrations provides the best agreement in the optical spectra with experiment (black line) and systems in which the coupling to high-frequency vibrations is reduced by a factor of 10 (green line) or completely removed (blue line).

The key conclusion is therefore that high-frequency vibrational modes and to a less extent the environmental noise intrinsic to the nature of such solvated supramolecular systems set a fundamental limitation on achieving fully coherent delocalization of the electronic wave function over multiple molecular sites. This finding is consistent with observations in other noisy supramolecular assemblies, such as photosynthetic complexes,^23^ molecular crystals,^7^ and π-conjugated polymers as those investigated in ref 71. In this work, Mannouch et al. demonstrate a clear separation of time scales between ultrafast exciton decoherence, driven by coupling to high-frequency vibrational modes, and the slower localization of the exciton density, which is induced by environmental dissipation and disorder. Reaching a perfectly coherent delocalized state would require eliminating all sources of disorder in the Hamiltonian coming from both fast and slow modes, an experimentally unfeasible condition, as it would necessitate rigidifying the molecular scaffold, drastically reducing structural flexibility, and preventing vibrations. Nonetheless, even if the electronic wave function is not fully coherent and coherences are rapidly damped, the excitation in such disordered aggregates remains delocalized over multiple sites. Such a delocalized equi-distribution of population across the aggregate’s units is fundamental, for instance, for enabling fast electronic transport, an effect that has been demonstrated for both excitons and charges in similar systems by several authors.^7,63,64,72,73^

## 4 Conclusions

Our work disentangles the long-standing ambiguity between exciton delocalization and quantum coherence in structurally and vibrationally noisy supramolecular aggregates. We investigated PDI self-assembled stacks in water solution, combining all-atom structural MD sampling with full quantum vibronic dynamics. The reliability of our *ab initio* model is validated by the excellent quantitative agreement obtained for the tetramer with experimental absorption spectra, accurately reproducing the blueshift, vibronic reshaping, and intensity redistribution upon aggregation. Our results reveal that optical features are governed not only by excitonic interactions but also by substantial mixing between LE and CT states. In particular, the higher density of CT states in the tetramer compared to smaller aggregates favors hybridization, redistributing oscillator strength and enabling rapid population transfer after photoexcitation.

Crucially, we address the dichotomy between delocalization and coherence. Even when the excitation is initially localized on a single site, the wave function rapidly spreads over the stacked monomers within tens of femtoseconds, as seen in inverse participation ratios as well as other measures (e.g., coherence length and total tangle) yielded by the analysis of the full electronic density matrix. Yet, both purity and total tangle reveal that quantum coherence decays on ultrafast time scales. Thus, our analysis demonstrates that delocalization, intended as equi-distribution of the state population on different molecular sites, survives, but coherence does not. Remarkably, we show that coupling to high-frequency vibrational modes alone is sufficient to induce decoherence, even without considering the disorder introduced by the slow fluctuations within the supramolecular aggregate and by the solvent.

This finding carries broad implications. Intrinsic vibrational noise, a universal feature of molecular systems, imposes a fundamental limit on coherent delocalization in soft materials. Nonetheless, the persistence of delocalized yet incoherent states still supports efficient exciton and charge dynamics, pointing to an important design paradigm: rather than pursuing hard-to-realize long-lived coherence, functional materials should exploit robust delocalization of the exciton population in noisy molecular environments.^7,64,72,73^ Beyond tackling the important delocalization–coherence debate, our work establishes a predictive protocol for exciton and charge dynamics in supramolecular aggregates, paving the way for experimental and theoretical studies on their photocatalytic, photovoltaic, and photothermal therapy applications.^74^ Looking ahead, incorporating relaxation of slower vibrational modes of both solute and solvent will be key to capturing the full hierarchy of time scales that govern exciton function in realistic molecular materials. Higher spin states could also be included in the dynamics to assess the role of other important processes, such as singlet fission.

## Supplementary Material



## Notes

Theoretical methods including: time-independent and time-dependent formulations, mixed quantum-classical Ad-MD|gLVC method, linear vibronic coupling Hamiltonian for extended aggregates, vertical gradients and hierarchical selection of modes, explanation of density matrix analysis, and various measurements of coherence and delocalization; computational details including: electronic structures and dynamics, constrained aggregate optimization, fragment diabatization approach, and ML-MCTDH settings; additional results including: static molecular spectrum, normal mode analysis and hierarchical selection of modes, Hamiltonian parameters, geometric analysis and correlation with electronic Hamiltonian parameters in the tetramer, analysis of environmental effects, convergence plots, comparison with and without CT, population evolution of dimer and trimer aggregates, and further analysis on coherence and excited state dynamics (PDF)

## b

Present Address: Department of Chemistry and Industrial Chemistry, University of Pisa, Via Giuseppe Moruzzi, 13, 56124 Pisa, Italy
