# Supplementary material for: Delocalization versus Coherence under Vibrational and Environmental Disorder in Photoexcited Supramolecular Aggregates
Source: J Am Chem Soc. 2026 Jan 19;148(3):3788–800. doi: 10.1021/jacs.5c20341 (PMC12856889; doi:10.1021/jacs.5c20341)
Supplement: Supplementary file 1 [file ja5c20341_si_001.pdf]

Electronic Supplementary Information:

Delocalization versus Coherence under Vibrational and Environmental Disorder in Photoexcited Supramolecular Aggregates

Samuele Giannini,[a,b,*] Alekos Segalina,[c] Daniele Padula,[d] Marta Cantina,[e] Mariachiara Pastore,[e,*] Giacomo Prampolini,[a] Fabrizio Santoro[a,*]

[a]Institute of Chemistry of OrganoMetallic Compounds, National Research Council (ICCOM-CNR), I-56124 Pisa, Italy;
[c]Center for Advanced Reaction Dynamics, Institute for Basic Science (IBS), Daejeon 34141, Republic of Korea; Department of Chemistry and KI for the BioCentury, Korea Advanced Institute of Science and Technology (KAIST), Daejeon, Republic of Korea
[d]Dipartimento di Biotecnologie, Chimica e Farmacia, Università degli Studi di Siena, Via A. Moro 2, 53100 Siena, Italy.
[e]Université de Lorraine & CNRS, LPCT, UMR 7019, F-54000 Nancy, France;
[b]Present Address : Department of Chemistry and Industrial Chemistry, University of Pisa, Via Giuseppe Moruzzi, 56124 Pisa, Italy.
Email: samuele.giannini@cnr.it; mariachiara.pastore@univ-lorraine.fr; fabrizio.santoro@cnr.it

Section S1: Theoretical methods

In this section, we briefly review the various strategies used in this work to simulate the steady-state absorption spectrum and the coupled electron-nuclear dynamics of PDI as isolated molecule and in its aggregated form considering the effects of nuclear vibrations, disorder and environmental effects.

Section S1.1: Time independent and time dependent formulations

Within the Born–Oppenheimer (BO) approximation framework, where electronic and vibrational degrees of freedom are separable, these states factorize as $|\Psi_f\rangle = |f\rangle|\boldsymbol{v}_f\rangle$. This separation enables the evaluation of spectral intensities based on the vibrational structure of both the ground and excited (adiabatic) electronic states. According to the well-known Fermi Golden Rule, the absorption intensity is proportional to the square of the transition dipole moment connecting the ground, $|\Psi_g\rangle = |g\rangle|\boldsymbol{v}_g\rangle$, and excited, $|\Psi_f\rangle = |f\rangle|\boldsymbol{v}_f\rangle$, electronic states, reflecting how efficiently light can induce the transition:

$$P_{|\Psi_g\rangle\rightarrow|\Psi_f\rangle} \propto |\langle\Psi_g|\hat{\mu}|\Psi_f\rangle|^2 = |\langle\boldsymbol{v}_g|\boldsymbol{\mu}|\boldsymbol{v}_f\rangle|^2 \quad \text{(S1)}$$

Where we have defined the transition dipole moment as $\boldsymbol{\mu} = \langle g|\hat{\mu}|f\rangle$.

The total spectrum is then given by the sum over all possible transitions, from all initial vibronic states (populated according to Boltzmann weights) to all final vibronic states which can be included as described in the next section. The lineshape of the absorption becomes:

$$L(\omega) = \sum_{\boldsymbol{v}_g}\sum_{\boldsymbol{v}_f} \rho_{\boldsymbol{v}_g}(T)\, |\langle\boldsymbol{v}_g|\mu_{gf}|\boldsymbol{v}_f\rangle|^2 \delta\left(\omega_f - \omega_g \pm (\Delta E/\hbar - \omega)\right) \quad \text{(S2)}$$

where $\rho_{v_g} = e^{-\beta\hbar\omega_i}/Z_{vib}$ is the the Boltzmann weight for the initial state and Z_{vib} the vibrational partition function in the initial state. δ is the Dirac delta function and selects the energy corresponding to the transition (arising from the resonance condition). Note that by including the ω prefactor in the previous equation, we obtain the absorption the spectrum, $\epsilon(\omega) = C_\epsilon \omega L(\omega)$, where C_ϵ is a constant that gathers all the physical constants (and depends on the kind of spectroscopy) and $\epsilon(\omega)$ is the absorption spectrum.[1] Note that the ± sign depends on the type of transition, being "+" for absorption and "−" for emission. Since the lineshape formula requires summing over all the vibronic states and requires knowledge of all the eigenvectors in frequency space, this formulation is referred to as Time-Independent (TI).

The equivalent expression can be obtained Fourier-transforming from the frequency to the time domain:

$$L(\omega) = \frac{1}{2\pi Z_{vib}} \int dt\ e^{\mp it(\Delta E/\hbar - \omega)} \sum_{v_g} \langle \boldsymbol{v}_g | \boldsymbol{\mu} e^{-i\widehat{H_f}\tau_f} \boldsymbol{\mu} e^{-i\widehat{H_g}\tau_g} | \boldsymbol{v}_g \rangle$$
$$= \frac{1}{2\pi Z_{vib}} \int dt\ e^{\mp it(\Delta E/\hbar - \omega)} \chi(t,T) \qquad \text{(S3)}$$

Where $\tau_f = t/\hbar$ and $\tau_g = -i\beta - t/\hbar$ and $\widehat{H_g}$ and $\widehat{H_f}$ are the Hamiltonians for initial and final states. From the expression above we can define a correlation between the initial and final states as $\chi(t,T) = \sum_{v_g} \langle \boldsymbol{v}_g | \boldsymbol{\mu} e^{-i\widehat{H_f}\tau_f} \boldsymbol{\mu} e^{-i\widehat{H_g}\tau_g} | \boldsymbol{v}_g \rangle = Tr\left[\boldsymbol{\mu} e^{-i\widehat{H_f}\tau_f} \boldsymbol{\mu} e^{-i\widehat{H_g}\tau_g}\right]$. Where we have introduced the trace of the operator, $Tr(\quad)$, which can be defined as the sum over all diagonal elements of the operator represented in a complete basis, which in this case is the set of the eigenvectors of the initial vibrational Hamiltonian. Details on this derivation are reported in Ref.[2,3]

When the potential energy surfaces are uncoupled and are approximated as harmonic around the equilibrium geometry, analytical formulations of both time-independent (TI) and time-dependent (TD) vibronic spectra become accessible and have been implemented in codes like our FCclasses3.[1,4] We have used the TI description to simulate the vibronic spectrum of the excited S1 state of isolated PDI molecule in Section S3.1.

When non-adiabatic couplings between electronic states become significant—such as in the case of excitonic bands in electronically coupled molecular aggregates—analytical expressions for the vibronic spectrum can no longer be derived. In such cases, an effective approach to compute the spectrum within the time-dependent (TD) framework is through the numerical propagation of the coupled electron-nuclear wavepacket over the interacting (diabatic) excited states using Quantum Dynamics (QD). In this work, these simulations are performed using the Multiconfigurational Time-Dependent Hartree (MCTDH) method, as implemented in the Quantics code.[5,6] The adiabatic electronic states of the system are reconstructed using a Linear Vibronic Coupling (LVC) Hamiltonian (or in the jargon of material scientists often called Frenkel-Holstein-type Hamiltonian), which is based on quadratic expansions of the diabatic potentials (d_k) and incorporates excitonic couplings between the states.[3] In this way, Eq. (S3) can be re-written in terms of auto- and cross-correlation functions between diabatic states as explained in Ref.[2,3,7]. In practice the TD approach requires the computation of correlation functions $\chi_{kl}(t) = \langle 0; d_k | \boldsymbol{\mu} e^{-i\hat{H}t} \boldsymbol{\mu} | d_l; 0 \rangle$, where $\boldsymbol{\mu} = \langle g | \hat{\mu}\, | d_l \rangle$ are the matrix elements of the electric dipole moment, 0 is the ground-vibrational state of the ground electronic state g, whose energy is set to 0. The general expression for the lineshape (at T=0° K) is finally retrieved as:

$$L(\omega) = \sum_{kl} \int_{-\infty}^{\infty} dt \, e^{i(E_{g0}+\hbar\omega)/\hbar t - \Gamma t^2} \; \chi_{kl}(t)$$
$$= \sum_{k} L_{kk}(\omega) + \sum_{k,l\neq k} L_{kl}(\omega) \quad \text{(S4)}$$

Note that, a quadratic damping term ruled by a parameter Γ was introduced, corresponding to a Gaussian broadening in the frequency domain. By construction, diabatic states are built to be virtually independent of the nuclear coordinates. This implies that the elements $\boldsymbol{\mu}$ can be considered constant (Condon approximation) and used to scale the time correlation functions computed via numerical propagation as explained in Ref.[2,3,7].

One drawback of full quantum dynamics is its high computational cost, which restricts its application to systems with a limited number of degrees of freedom (DoFs). Here, we address this issue by employing an effective selection of the most relevant modes to be included in the Hamiltonian, as explained in Section S1.3. Another important challenge is the treatment of anharmonic intermolecular vibrations—often characterized by low frequencies— as well as the influence of the environment and their contribution to the overall energetic disorder when considering the dynamics of the molecular aggregate. We address these challenges below.

Section S1.2: Mixed quantum-classical Ad-MD|gLVC method

Mixed Quantum Classical (MQC) strategies provide a cost-effective, strategy not only to include low frequency vibrations of the of molecular aggregate while accounting for important vibronic effects, but also to include the contributions of the environment. Some of us recently introduced a MQC approach named Ad-MD|gLVC.[7] This protocol partitions the coordinates of the entire system (aggregate + medium) into soft $(\boldsymbol{R}_{\mathrm{lf}})$ and stiff (**r**) coordinates as discussed in the main text. The soft and slow DoFs $(\boldsymbol{R}_{\mathrm{lf}})$ of the aggregate and solvent modes are included through classical molecular dynamics (MD) sampling performed with quantum-mechanically-derived force fields (QMD-FF) as described in Section S2, while the stiff modes are described at the vibronic level using harmonic potentials specific of the molecule in its excited state (locally excited, cationic or anionic state as described in Section S3 depending on whether localized excitations or charge transfer states are considered).

The final Ad-MD|gLVC spectrum is obtained by averaging the signals computed at the quantum mechanical (QM) level for the stiff coordinates over the configurations collected from the MD trajectory including the effect of the solvent as:

$$L(\omega) = \frac{1}{N_{\mathrm{snap}}} \sum_{s} L_s^{\boldsymbol{q}^r}(\omega) \quad \text{(S5)}$$

where the sum is along the different snaphots (s) sampled along MD. Each spectrum $L_s^{\boldsymbol{q}^r}$, is computed in reduced-dimensionality normal-coordinates space $\boldsymbol{q}^r$ obtained by projecting out all the flexible coordinates $\boldsymbol{R}_{\mathrm{lf}}$. As explained in more details in Section S3, $\boldsymbol{q}^r$ are obtained for the optimum ground state structure of the isolated molecule. The potential energy surfaces of the molecule in its ground or excited (cationic, anionic, locally excited) states constructed along $\boldsymbol{q}^r$ are assumed to be harmonic.

Section S1.3: Linear Vibronic coupling Hamiltonian for extended aggregates

The linear vibronic coupling Hamiltonian is written as a sum of electronic, vibrational and electron-vibronic Hamiltonians (see also Fig. S1):

$$\hat{H} = \hat{H}^{el} + \hat{H}^{vib} + \hat{H}^{el-vib} \quad \text{(S6)}$$

The electronic Hamiltonian ($\hat{H}^{el}$) describes the various types of excitations within the aggregate—namely, localized excitations (LE) on individual molecules and inter-molecular charge transfer (CT) excitations—as well as their pairwise interactions. This Hamiltonian is evaluated for a given snapshot sampled along the slow degrees of freedom ($\boldsymbol{R}_{\mathrm{lf}}$), following the MQC strategy. It can be constructed either by computing each matrix element individually (as done, for instance, in Refs.[8,9]), or directly via a diabatization technique applied to the full aggregate.[7] In this work, we adopted the latter approach and the details for the protocol are described in Section S2. Note that, we also included environmental effects using a QM/MM like approach. $\hat{H}^{el}$ can be further divided into different blocks to classify the interactions of different nature:

$$\hat{H}^{el} = \hat{H}_{\mathrm{LE}}(\boldsymbol{R}_{\mathrm{lf}}) + \hat{H}_{\mathrm{CT}}(\boldsymbol{R}_{\mathrm{lf}}) + \hat{H}_{\mathrm{LE-CT}}(\boldsymbol{R}_{\mathrm{lf}}). \quad \text{(S7)}$$

The block of LE states describes the interactions between bound electron-hole pair residing on the same molecular site k and can be written as

$$\hat{H}_{\mathrm{LE}} = \sum_{k} E_k^{LE}(\boldsymbol{R}_{\mathrm{lf}})|e_k\rangle\langle e_k| + \sum_{k,l} V_{kl}|e_k\rangle\langle e_l|. \quad \text{(S8)}$$

Here, the energy of a local Frenkel exciton ($|e_k\rangle$), is indicated with E_k^{LE} and it represent the vertical energy of a localized excitation. The off-diagonal elements of this block are the excitonic couplings, V_{kl}, which describe the interactions between tightly bound electron-hole pair sitting either on molecules k or l and allow for excitation energy transfer between the two.

The second term H_{CT} represents the charge transfer Hamiltonian which describe the interactions between charge transfer excitons. This is written as:

$$\begin{aligned}\hat{H}_{\mathrm{CT}} = &\sum_{k,s\neq 0} E_{k,l}^{CT}(\boldsymbol{R}_{\mathrm{lf}})|\mathrm{c}_k,\mathrm{a}_{k+s}\rangle\langle \mathrm{c}_k,\mathrm{a}_{k+s}| \\ &+ \sum_{k,s\neq 0,s'\neq 0} t_e(\boldsymbol{R}_{\mathrm{lf}})|\mathrm{c}_k,\mathrm{a}_{k+s}\rangle\langle \mathrm{c}_k,\mathrm{a}_{k+s'}| \\ &+ \sum_{k,s\neq 0,s'\neq 0} t_h(\boldsymbol{R}_{\mathrm{lf}})|c_{k+s},a_k\rangle\langle c_{k+s'},a_k|\end{aligned} \quad \text{(S9)}$$

where the $|c_k,a_{k+s}\rangle$ denotes the CT exciton where the hole (cationic state) is located on the site k, while the electron (anionic state) is on molecule l located at distance s from the first molecule. The vectors s and s' denote the electron/hole separation (in molecular units) and are restricted to nonzero values. When $s = 0$ the electron and hole are located on the same molecule resulting in a LE excitation. $E_{k,l}^{CT}(\boldsymbol{R}_{\mathrm{lf}})$ is the energy of the charge transfer state as shown in the main text increases with increasing electron/hole distance and depends on the environmental response. CT excitons couple to one another via electron and hole transfer integrals, t_{e} and t_{h}, respectively. The charge transfer integrals, which to a good approximation are a result of the interactions between either the HOMO orbitals or the LUMO orbitals of two interacting chromophores are influenced by the extent of spatial overlap between them.[8,10] Consequently, the coupling decreases exponentially as the distance between the chromophores increases, see Fig. 2 main text.

The mixing between FE and CT excitons is given by the Hamiltonian block $H_{\mathrm{LE-CT}}$ which is written as:

$$\hat{H}_{\text{LE-CT}} = \sum_{k,s\neq 0} D_e(\boldsymbol{R}_{\text{lf}})|e_k\rangle\langle c_k, a_{k+s}| + h.c. \\ + \sum_{k,s\neq 0} D_h(\boldsymbol{R}_{\text{lf}})|e_k\rangle\langle c_{k+s}, a_k| + h.c. \tag{S10}$$

where $D_e(\boldsymbol{R}_{\text{lf}})$ and $D_h(\boldsymbol{R}_{\text{lf}})$ are the photoinduced electron and hole transfer interactions, respectively, which couple FE and CT states, and where $h.c.$ stands for the Hermitian conjugate. This Hamiltonian block is crucial because it allows dark CT states to borrow oscillator strength from bright LE. This, in turn, significantly impacts the optical properties and excited state dynamics of the aggregate. It is worth noticing that as carefully described in Ref.[11], $D_e(\boldsymbol{R}_{\text{lf}})$ and $D_h(\boldsymbol{R}_{\text{lf}})$ are not strictly the same as t_e and t_h, since additional exchange-like terms appear in the expression of the former couplings. However, the leading contribution in the calculation of $D_e(\boldsymbol{R}_{\text{lf}})$ and $D_h(\boldsymbol{R}_{\text{lf}})$ is related to the one-electron terms (Fock matrix elements) involving the interaction of LUMO orbitals for the former and HOMO orbitals for the latter as it is the case for t_e and t_h. Consequently, these terms decay exponentially with the distance between donor and acceptor sites as well and are very similar.

All the matrix elements of the Hamiltonian can be found through a diabatization procedure applied for each MD step as explained in Section S2.

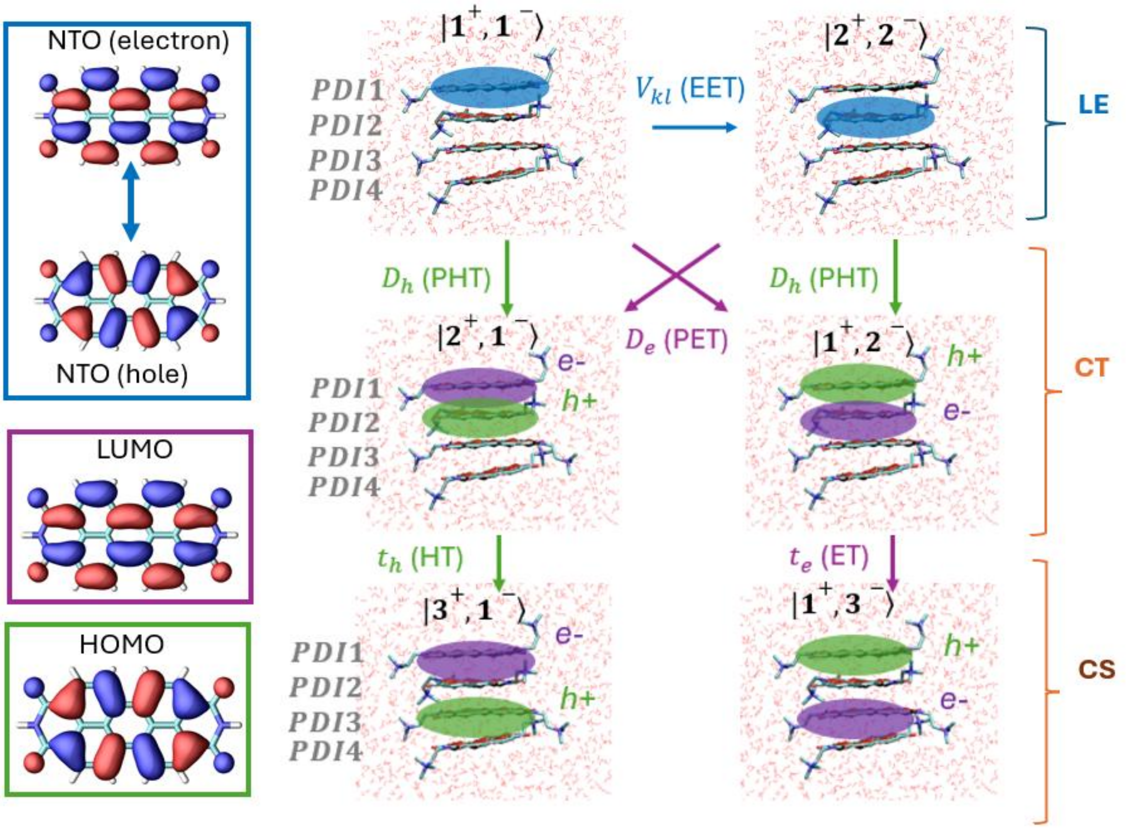

Figure S1: Schematic representation of the different processes and steps describe by the electronic Hamiltonian $\hat{H}^{el}$ in Eq. S7. The coupling between localized Frenkel-type excitons (V_{kl}) is represented in blue (yielding excitation energy transfer (EET)). Photoinduced electron transfer (PET) and photoinduced hole transfer (PHT) processes responsible for exciton splitting and promoted by D_e and D_h electronic interactions are shown in magenta and green respectively. Finally, electron and hole transfer processes with transfer integrals t_e and t_h, respectively with the same color code as the photoinduced process to show that and electron or the hole are moving. The orbitals involved in the LE and CT states are also represented with the same color code (left panels).

The electronic excitations—i.e., the formation of anionic, cationic, and excitonic states—are strongly coupled to high-frequency vibrational modes. This vibronic interaction is, for instance, responsible for the pronounced vibronic progression observed in the single-molecule spectra discussed in Section S3. These fast degrees of freedom are incorporated into the Hamiltonian by considering a reduced-dimensionality space of normal coordinates, $\boldsymbol{q}^r$, obtained by projecting out the slow and flexible coordinates $\mathbf{R}_{\mathrm{lf}}$:

$$\hat{H}^{el-vib} = \hat{H}^{LE-vib} + \hat{H}^{CT-vib} \tag{S11}$$

The first term represents the coupling between LE states and the nuclear degrees of freedom is:

$$\hat{H}^{LE-vib} = \sum_{k} \sum_{\alpha}^{Nexc} \lambda_k^{\alpha,LE} q_\alpha \, |LE_k\rangle\langle LE_k| \tag{S12}$$

Similarly, the coupling between CT states and the nuclear degrees of freedom is given by:

$$\begin{aligned}\hat{H}^{CT-vib} = &\sum_{k,s\neq 0} \sum_{\alpha}^{Nanion} \lambda_{k+s}^{\alpha,a} q_\alpha \, |\mathrm{c}_k, \mathrm{a}_{k+s}\rangle\langle \mathrm{c}_k, \mathrm{a}_{k+s}| \\ &+ \sum_{k,s\neq 0} \sum_{\alpha}^{Ncation} \lambda_k^{\alpha,c} q_\alpha \, |c_k, \mathrm{a}_{k+s}\rangle\langle \mathrm{c}_k, \mathrm{a}_{k+s}| \end{aligned} \tag{S13}$$

$\lambda_k^{\alpha,(LE,a,c)}$ represent the gradients of the excited potential energy surface (excitonic, cationic or anionic) at the equilibrium geometry of the ground state and are referred to as first-order intra-state electronic-vibrational coupling constants (which related also to the dimensionless shift g_k^α by $\lambda_k^{\alpha,} = g_k^\alpha \hbar\omega_\alpha$).[12]

Note $\hat{H}^{LE-vib}$ are $\hat{H}^{CT-vib}$ are similar to the Hamiltonians used in Ref.[13,14], with the noticeable difference that in this work the vibronic part of the Hamiltonian includes multiple degrees of freedom coupled to the excitation as exemplified by the sum over α (not just a single effective mode as it was done in Ref.[8,13,14]) and the effect of the complex morphology of the system is fully taken into account. Moreover, in this work, such q_α modes are selected with a powerful hierarchical procedure discussed in Section S1.4.

The kinetic and potential components of the vibrational Hamiltonian $\hat{H}^{vib}$ are written in terms of the *M* (mass-weighted) dimensionless coordinates q_α and their conjugate momenta p_α as:

$$\hat{H}^{vib} = \hat{K}^{vib} + \hat{V}^{vib} = \frac{1}{2}\sum_{\alpha} \omega_\alpha \, p_\alpha^2 + \frac{1}{2}\sum_{\alpha} \omega_\alpha \, q_\alpha^2 \tag{S14}$$

Where ω_α is the frequency of the specific hierarchically selected mode α (considering $\hbar = 1$). Note that a dimensionless coordinate q_α and related momenta p_α are found from the analogue quantity with dimensions by: $q_\alpha = \sqrt{\frac{\omega_\alpha}{\hbar}}\sqrt{m}x_\alpha$ and $p_\alpha = \frac{mv_\alpha}{\sqrt{m\omega\hbar}}$, respectively.

Section S1.4: Vertical gradients and Hierarchical selection of modes

We remark here that to make the description of the aggregate's vibronic states computationally tractable; we follow the "simplified" route introduced in Ref.[7]. Specifically, we assume that (i) the intermolecular excitonic interactions are only modulated by the slow $\boldsymbol{R}_{\mathrm{lf}}$ coordinates and independent of the fast ones, and (ii) the vibronic structure (i.e., the diagonal gradients λ_k^{α} in Eq. S12,S13) is independent of the specific snapshot, s. These are well-justified approximations, as excitonic interactions are primarily modulated by low-frequency intermolecular modes—whose influence is accounted for in the time-dependent modulation of the electronic Hamiltonian along the MD trajectory (see Section S2) and the vibronic substructure is essentially dictated by fast modes that are locally and instantaneously rearranging the to the configuration of the slow modes.

In practice, under the above approximations, we estimate the gradients λ_k^{α} appearing in Eq. S12 and S13 using the structure of an isolated PDI monomer optimized in its ground state in the gas phase. A (quantum) harmonic oscillator model is used to approximate excited and ground states potential energy surfaces (PES) as implemented in the FCclasses3.0 software.[1,4]. In particular, the ground state PES is simply obtained using QM hessian and frequencies at the optimized PDI geometry. For the excited-state PES, which in this case refer to locally excited, cation and anion of the molecule, among the several available models,[1,4] we adopt the Vertical Gradient (VG) approach. Considering that the final-state PES has the same normal modes and frequencies as the initial-state, VG only accounts for the effect of the (dimensionless) displacement, g_{α}, from the equilibrium position that is estimated with a harmonic model from the excited vertical gradient at the ground state geometry. The displacement for each mode, in turn, determines the relaxation energy of such a mode ($\lambda_{\alpha}^{rel} = \frac{g_k^{\alpha^2}}{2}\hbar\omega_{\alpha}$). The gradients of a localized state indicated with $\lambda_k^{\alpha,LE}$ are retrieved with a single point TD-DFT calculation. While the gradients of the CT states can be constructed using the gradients of the cationic and anionic states ($\lambda_{k+s}^{\alpha,a}$ and $\lambda_k^{\alpha,c}$) obtained again for the optimized molecule in its ground state in the gas phase.

Notably, considering all the gradients along all the normal modes of each diabatic state is still too demanding when analysing extended aggregates. Thus, to reduce the number of modes to include in the Hamiltonian in Eq. S6, we adopted a Hierarchical effective mode selection (HEMS), analogously to what was done in Ref.[7] Interestingly, the low- to medium-resolution spectrum can be efficiently computed using a hierarchical transformation of the Hamiltonian, structured into blocks of sequentially coupled effective modes.[15,16] It has been demonstrated that the use of these effective modes ensures that reduced-dimensionality models can still replicate the dynamics of the full-dimensional system for a short time.[17–19] This hierarchy is constructed from the practical point of view through a generalized Lanczos as explained in Ref. [15].

Section S1.5: Density matrix

Considering a generic quantum state $|\Psi\rangle$, the density matrix is defined as:

$$\hat{\rho}(t) = |\psi(t)\rangle\langle\psi(t)| \tag{S15}$$

Adopting the complete basis set we can write the density matrix as

$$\hat{\rho} = \sum_{nm} |\phi_n\rangle\langle\phi_n|\psi\rangle\langle\psi|\phi_m\rangle\langle\phi_m| = \sum_{nm} \rho_{mn}(t)|\phi_n\rangle\langle\phi_m| \tag{S16}$$

The density matrix, $\boldsymbol{\rho}$, with elements ρ_{mn} is Hermitian. Thus, the matrix of its eigenvectors **U** is unitary $\boldsymbol{U}^{\dagger}\boldsymbol{U} = \mathbf{1}$. Imagining having a vibronic wavefunction $|\psi\rangle = |\Psi(t)\rangle|\boldsymbol{v}\rangle$, we can calculate the density matrix by tracing out the nuclear degrees of freedom, thus the density matrix will become an electronic density matrix:

$$\boldsymbol{\rho}_{el}(t) = Tr_v \boldsymbol{\rho}(t) \tag{S17}$$

Adopting the hole-electron particle basis we can write the matrix representation of the electronic density matrix $\boldsymbol{\rho}_{el}(t)$ as:

$$\begin{aligned} \hat{\rho}_{el} &= \sum_{|k,l\rangle} \sum_{|m,n\rangle} \langle k,l|\Psi(t)\rangle\langle\Psi(t)|m,n\rangle \, |k,l\rangle\langle m,n| \\ &= \sum_{|k,l\rangle} \sum_{|m,n\rangle} u_{kl}(t) u_{mn}^{*}(t) |k,l\rangle\langle m,n| \\ &= \sum_{|k,l\rangle} \sum_{|m,n\rangle} \rho_{kl,mn}(t) |k,l\rangle\langle m,n| \end{aligned} \tag{S18}$$

Section S1.6: Purity of a state

The purity of a general density matrix can be defined as the trace of the square of the density matrix.

$$\gamma = Tr(\boldsymbol{\rho}^2) \tag{S19}$$

And for a generic state we have $Tr(\boldsymbol{\rho}^2) \leq Tr(\boldsymbol{\rho})^2$. A pure state can always be written such that $Tr(\boldsymbol{\rho}^2) = Tr(\boldsymbol{\rho})^2$, while for a mixed state this is not possible. For an aggregate with N states, its maximum and minimum values are 1 for a pure state and $1/N$ for an equally populated but completely incoherent mixed state.

Section S1.7: Population analysis

Considering our hole-electron basis set, we can express the population of hole, electron and exciton using the following projections of the wavefunction on the basis set functions.

The population of the hole wavefunction becomes:

$$P_k^{(h)}(t) = \sum_{l}^{N} |\langle k,l|\Psi(t)\rangle|^2 = \sum_{l}^{N} |u_{kl}|^2 = \sum_{l}^{N} \left|\rho_{kl,kl}\right|^2 \tag{S20}$$

For the electron population we have:

$$P_l^{(e)}(t) = \sum_{k}^{N} |\langle k,l|\Psi(t)\rangle|^2 = \sum_{k}^{N} |u_{kl}|^2 = \sum_{k}^{N} \left|\rho_{kl,kl}\right|^2 \tag{S21}$$

The exciton population in the subset of the excitonic states becomes:

$$P_k^{(exc)}(t) = \frac{1}{N_{exc}} \sum_{k}^{N_{exc}} |\langle k, k|\Psi(t)\rangle|^2 = \frac{1}{N_{exc}} \sum_{k}^{N_{exc}} |\rho_{kk,kk}|^2 \quad \text{(S22)}$$

Note that these values can be computed for each QD trajectory and then the average can be taken such as: $\left\langle P_k^{(particle)}(t) \right\rangle_{N_{traj}}$, where the angular parentheses indicate an ensemble average.

Section S1.8: Inverse Participation ratio

A common measure of delocalization is the inverse participation ratio (IPR)[20,21].

$$IPR = \frac{\left(\sum_{k,l}^{N} |\rho_{kl,kl}|\right)^2}{\sum_{k,l}^{N} |\rho_{kl,kl}|^2} = \frac{\left(\sum_{i}^{N} |\rho_{i,i}|\right)^2}{\sum_{i}^{N} |\rho_{i,i}|^2} \quad \text{(S23)}$$

Where in the last expression, we have joined the indices of the density matrix.

The IPR ranges from 1 for a fully localized state to N for a delocalized one. Often the IPR is indicated as reversed (i.e. 1/IPR, and this goes from 1/N for a fully localized state to 1 for a fully delocalized one). It should be noted that IPR was introduced for pure states,[22] and is dependent on the basis set in which it is expressed. Note that in some publications Eq. S23 is called participation ratio (PR). We prefer to call it Inverse participation ratio (IPR).

In summary:

$$IPR = \begin{cases} 1 & i)\text{: localized if } \rho_{ii}{=}1,\ \rho_{jj}{=}0\ \forall j \neq i,\ \rho_{ij}{=}0\ i \neq j \\ N & ii)\text{: equi-distributed incoherent if } \rho_{ii}{=}1/\text{N}\ \forall i,\ \rho_{ij}{=}0\ i \neq j \\ N & iii)\text{: fully delocalized if } \rho_{ij}{=}1/N\ \forall i,j \end{cases}$$

This measure is dependent on the basis set in which it is expressed, hence for holes and electrons, we have respectively:

$$IPR^{(h)} = \frac{P_{tot}^{(h)}}{\sum_k |P_k^{(h)}(t)|^2} \quad \text{(S24)}$$

and

$$IPR^{(e)} = \frac{P_{tot}^{(e)}}{\sum_l |P_l^{(e)}(t)|^2} \quad \text{(S25)}$$

The IPR can only measures how much the system is equally distributed on the different basis sets, without bringing any information on its coherence (related to the off-diagonal elements of the density matrix).

Section S1.9: Coherence length

In order to consider also the coherences, the off-diagonal elements of the density matrix need to be included. In Ref.[23] the authors proposed to adopt a generalized definition of IPR, that was named coherence length (CL),[21]

$$CL = \frac{\left(\sum_{kl,mn}|\rho_{kl,mn}|\right)^2}{N\sum_{kl,mn}|\rho_{kl,mn}|^2} = \frac{\left(\sum_{i,j}|\rho_{i,j}|\right)^2}{N\sum_{i,j}|\rho_{i,j}|^2} \qquad \text{(S26)}$$

In which in the last expression we simplified the index and wrote the equation in analogy with the IPR.

N is introduced here as a normalization factor, so that CL has the same maximum as the IPR. Like the IPR, CL also depends on the basis set adopted for defining ρ. To appreciate the different information content of the two quantities, let us compute the limiting values of CL for three different situations: i) a pure state fully localized on a single member of the basis set, ii) a fully incoherent mixed state equally distributed over all states of the basis set, and iii) a fully delocalized, fully coherent pure state. In summary:

$$CL = \begin{cases} 1/N & i)\text{: localized if } \rho_{ii}{=}1,\ \rho_{jj}{=}0\ \forall j \neq i,\ \rho_{ij}{=}0\ i \neq j \\ 1 & ii)\text{: equi-distributed incoherent if } \rho_{ii}{=}1/\text{N}\ \forall i,\ \rho_{ij}{=}0\ i \neq j \\ N & iii)\text{: fully delocalized if } \rho_{ij}{=}1/n\ \forall i,j \end{cases}$$

Section S1.10: Total Tangle

Using the purity and the IPR we can use their "difference" to quantify the amount of entanglement.[21]

$$E_T(\rho) = 2\sum_{i,j>i}^{N}|\rho_{i,j}|^2 = [Tr(\rho^2) - IPR^{-1}] \qquad \text{(S27)}$$

In physical terms, a large IPR^{-1} (indicating that the excitation is relatively localized) corresponds to a low degree of entanglement. This highlights once more the close link between coherence in the form of exciton delocalization and entanglement in the system. With the definition in Eq. S27 the total tangle maximum value for a fully coherent state is 1-1/N. Note that some texts include a factor 2 in the definition of Eq. S27 and for a fully coherent 2(1-1/N), instead.

$$E_T(\rho) = \begin{cases} 0 & i)\text{: localized if } \rho_{ii}{=}1,\ \rho_{jj}{=}0\ \forall j \neq i,\ \rho_{ij}{=}0\ i \neq j \\ 0 & ii)\text{: equi-distributed incoherent if } \rho_{ii}{=}1/\text{N}\ \forall i,\ \rho_{ij}{=}0\ i \neq j \\ 1-1/N & iii)\text{: fully delocalized if } \rho_{ij}{=}1/N\ \forall i,j \end{cases}$$

Section S1.11: Von Neumann entropy

The Von Neumann entropy is defined as

$$S_{VN} = -Tr(\boldsymbol{\rho} ln \boldsymbol{\rho}) \qquad \text{(S28)}$$

The minimum (for a pure state) and maximum (for a fully incoherent state with equal populations) values are $0 \leq S_{VN} \leq ln(N)$. Interestingly, the von Neumann entropy depends only on the eigenvalue spectrum of the density matrix, not on how it is represented in any particular basis.

Section S1.12: Relative entropy

For excitonic systems including only a single excitation, Sarovar et al.[24] proposed to use relative entropy as a measure of global entanglement

$$E[\rho] = -Tr\left(\rho_{i,i} ln(\rho_{i,i})\right) - S_{VN} \tag{S29}$$

where S_{VN} is the von Neumann entropy. By construction $E[\rho] \geq 0$ and it vanishes for separable states. This quantity corresponds to the relative entropy of entanglement, defined as the minimum relative entropy distance between the state $\rho$and the set of separable states. In the single-excitation manifold, this minimum is attained for the separable state that shares the same diagonal elements (populations) as ρ. This state is therefore identified as the closest separable state to ρin the sense of relative entropy.

The limiting values for our three cases are:

$$E(\rho) = \begin{cases} 0 & i)\text{: localized if } \rho_{ii}=1,\ \rho_{jj}=0\ \forall j \neq i,\ \rho_{ij}=0\ i \neq j \\ 0 & ii)\text{: equi-distributed incoherent if } \rho_{ii}=1/\text{N}\ \forall i,\ \rho_{ij}=0\ i \neq j \\ 1-1/N & iii)\text{: fully delocalized if } \rho_{ij}=1/N\ \forall i,j \end{cases}$$

Section S1.13: State character analysis

Fractional LE and CT character of *n*th eigenstate can be obtained by diagonalizing the electronic Hamiltonian (Eq. S7) and using the eigenvectors to determine the amount of mixing. In particular,

$$\nu_{CT}^{n} = \sum_{i \neq j}^{N} \sum_{j}^{N} \left|U_{ij}^{n}\right|^2 \tag{S30}$$

And

$$\nu_{FE}^{n} = \sum_{i}^{N} |U_{ii}^{n}|^2 \tag{S31}$$

Note that the same analysis could be done with the vibronic Hamiltonian if its eigenstates were accessible in the electron-hole basis.[8]

Section S2: Computational details

Section S2.1: Electronic structure and dynamics

All quantum mechanical (QM) calculations were performed at the DFT and TD-DFT levels using the Gaussian 16 package, with the CAM-B3LYP functional and the 6-31G(d) basis set as done in previous work,[7] including Grimme's D3 dispersion correction. We note that the use of a larger basis set would be computationally very demanding since, as explained below, our approach requires constrained structural optimizations followed by the calculation of multiple excited states to achieve a complete diabatization.

In the case of aggregated systems, to perform the sampling of the electronic Hamiltonian and include energetic disorder, molecular dynamics trajectories taken from Ref.[25] were used. From these we extracted uncorrelated snapshots every 10 ps depending on the system. The quantum-mechanically derived force-field (FF) used to generate such trajectories has been previously parametrized and validated by some of us in Ref.[7,26] using QM data obtained using the aforementioned long-range corrected hybrid functional.

Section S2.2: Constrained aggregate optimization

Following the approach used in Ref.[7] for the dimer, we constructed vibronic LVC Hamiltonians of the different aggregates in the subspace of the fast degrees of freedom only, while accounting for the dynamics of the slow coordinates using the MD statistical sampling. To this end, for each MD snapshot, we performed a DFT geometry re-optimization of each individual PDI monomer within the aggregate. In particular, only the atoms belonging to the core of a given PDI core were allowed to relax, while atoms in the lateral pendants and in the other PDI monomers were kept fixed. An ONIOM QM/MM scheme was adopted: chemical species within a 4 Å radius (water, Cl^-, and the other PDI) were included in the DFT optimization at the MM level, using QMD-FF point charges and Lennard-Jones terms. All other species within a 20 Å radius were treated as fixed QMD-FF point charges. Finally, for each snapshot, the optimized geometry of the dimer, trimer and tetramer were straightforwardly obtained by combining the two, three or four optimized monomers, respectively. An example of the ONIOM QM/MM partition is given in Fig. S2, where we have indicated the different layers with different representations.

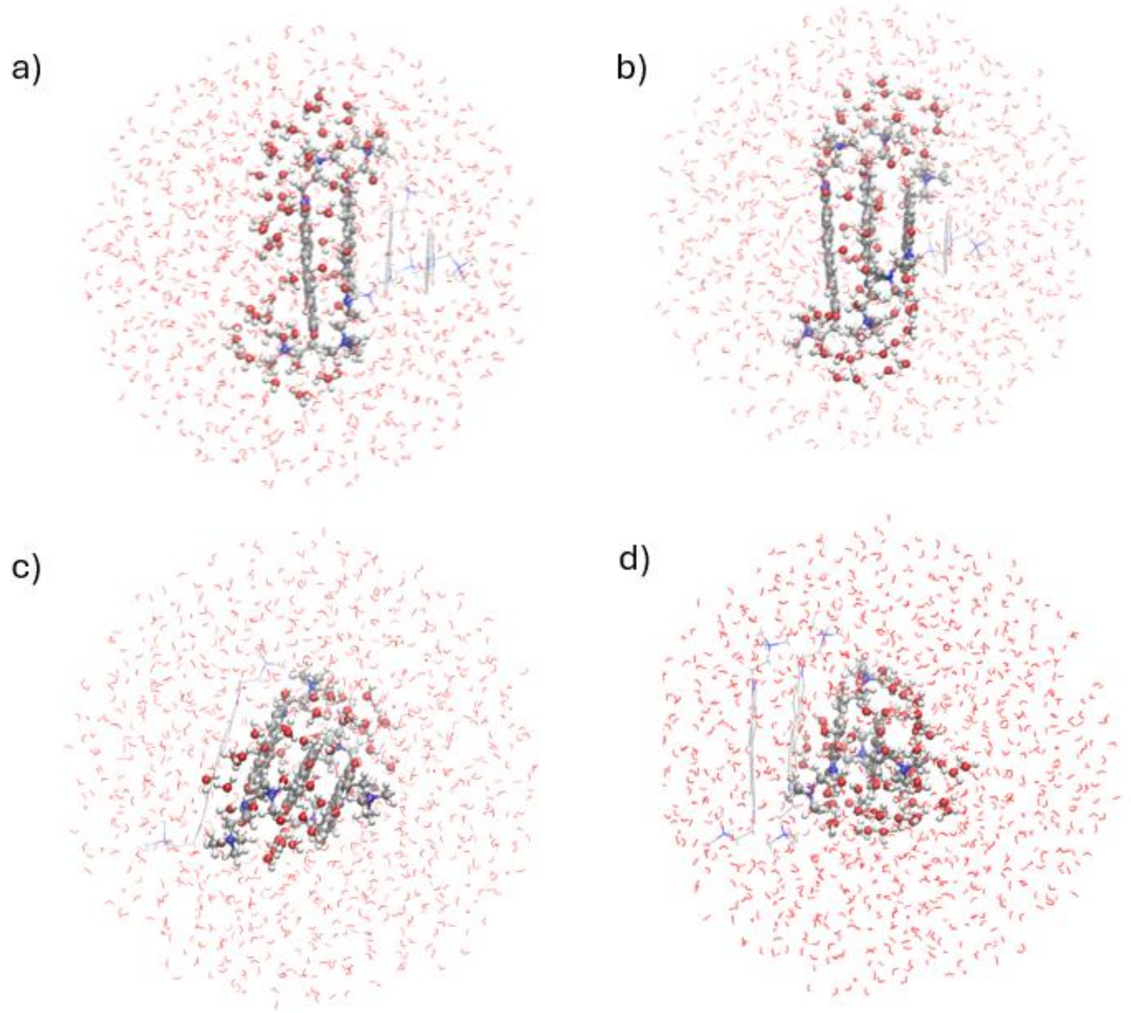

Figure S2: Representation of the various layers characterizing the ONIOM QM/MM approach used for the description of tetramer in solution. A PDI molecule is included in the QM region together with explicit species (water, Cl- and PDI) within 4.0 Å from the given PDI center of mass (Corey–Pauling–Koltun (CPK) and related colour scheme are used). The other species within 20 Å from. All the rest of the molecules within 20 Å around the investigated PDI are treated as atomic point charges (PC) placed at the different atomic positions extracted from the MD. Each panel in a), b), c) and d) represents the QM PDI number 1, 2, 3, 4, respectively.

Section S2.3: Fragment diabatization

The electronic Hamiltonian $\widehat{H}^{el}$ is constructed using our diabatization approach (through the open-source OVERDIA software), developed by some of the authors.[27] This method performs an adiabatic-to-diabatic transformation based on a maximum overlap criterion.[27] It takes as input the adiabatic states from TD-DFT calculations carried out with Gaussian 16,[28] along with definitions of the reference (localized) states used in the transformation. The reference quasi-diabatic states can be defined in several ways; here, we consider the states of the individual monomers forming the aggregate at their Franck–Condon (FC) geometries.

All monomer and aggregate calculations employed a two-layer approach to account for the environment, incorporating electrostatic embedding (EE) of all solvent molecules and counterions within a 20 Å radius. These were included as QMD-FF point charges (pc). In the monomer calculations, the electrostatic effect of the second monomer on the reference states was included by treating it as part of the EEpc shell (see Fig. S2). Reference states for the LE states were obtained by reading the

corresponding TD-DFT response vectors from the monomer calculations. For CT states, reference states were defined as orbital transitions from the HOMO of one monomer to the LUMO of the other, and vice versa.[7] To account for the effect of structural disorder, the diabatization was repeated for each configuration of the aggregate extracted along MD (and partially optimized) to make sure that the electronic Hamiltonian depends only on $\boldsymbol{R}_{\mathrm{lf}}$. About 60 adiabatic states of the aggregate are computed at each snapshot to have a complete diabatization.

The diabatization procedure enables the extraction of all the matrix elements (excitation energies and their excitonic couplings) in Eq. S7. For completeness, we note also that similar diabatization schemes based on molecular properties have been used by some of the authors in other works[8]. We tested that these different schemes give essentially the same results as the OVERDIA approach used here. We note in passing, that the sparsity of the Hamiltonian can be exploited to efficiently construct the full matrix without resorting to computationally expensive diabatization evaluations of all couplings.[8,9] Finally, we note that since the excitonic couplings are directly computed from a diabatization that consistently consider reference states with the same phase, the important coupling-sign relationship is fulfilled. See Ref.[8] for an extensive discussion on this issue.

Section S2.4: MCTDH settings

Nuclear wave-packet propagations were carried out using the MCTDH method[29–33], as implemented in the QUANTICS code.[5] The wavepackets were propagated for 200 fs in steps of 0.5 fs. We adopted the variable mean field scheme with a Runge-Kutta integrator of order 5 and an accuracy threshold of 10^{-7}.

When the number of coordinates is relatively small, we run standard MCTDH wavepacket propagations using with constant mean field scheme with an accuracy of 10^{-5}. A Bulirsch-Stoer extrapolation integrator was adopted for the SPFs with a maximal order of 7 and 10^{-5} accuracy, while a SIL integrator was exploited for the A vector with maximal order of 5 and accuracy of 10^{-5}.

Notably, however, the PDI molecule already possesses 112 vibrational coordinates, which makes a straightforward application of MCTDH for large aggregates computationally prohibitive. To address this, fully converged low-resolution spectra that account for all nuclear degrees of freedom were obtained by employing a hierarchical representation of the Hamiltonian in terms of effective collective coordinates as well as a multi-Layer (ML) extension of MCTDH.[33] In the hierarchical representation (see Section S1.4, the coordinates were grouped into blocks, each containing several modes, defined so that the short-time dynamics—relevant for the low-resolution spectrum—are dominated by only a few blocks. Fig. S10 shows that using just two blocks (12 coordinates for a dimer, 18 for a trimer and 24 for a tetramer, respectively) are sufficient to produce converged spectra for a Gaussian broadening with a half-width at half-maximum (HWHM) of 0.032 eV and adding a third block leads to only minor differences. Therefore, in the calculations of the main text two blocks were used to compute the spectra and population dynamics.

In the cases where ML-MCTDH was used, we did it with a 5th order Runge-Kutta integration and the mean field matrices were updated at each integration step (Variable Mean Field, VMF). The same ML-MCTDH settings were used for the computation of the time-evolution of the populations. Different ML trees were checked to be sure to reach convergence.

Section S2.5: Ad-MD|gLVC workflow for simulating molecular aggregates

The workflow employed in this work follows these steps:

1) Computation of the vibronic structure of the molecule (performed only once) for the optimized geometry of the molecule. Validation by comparing the molecular spectrum again the experiment and hierarchical selection as detailed in Section S2.
2) Extraction of snapshots from classical MD performed with QMD-FF as described above.
3) Calculation of the electronic Hamiltonian using the diabatization tool Overdia to calculate all diagonal and off-diagonal matrix elements of dimer, trimer and tetramers accounting for the explicit electrostatic effect of the environment.
4) Construction of the LVC Hamiltonian and operator file for quantum dynamics simulations performed on the different snapshots characterized by a different static disorder.
5) Calculation of the spectrum of the molecule using Eq. S4 and excited state population dynamics and delocalization.

Section S3: Additional Results

Section S3.1: Static molecular spectrum

The spectrum of a single PDI molecule, used for reference purposes, was computed in this work using the so-called static approach. That is, evaluating the excitation energy of the bright S1 state of the molecule for a single conformer taken at the minimum optimized geometry. The optimization in this case was performed by removing the side chains at CAM-B3LYP/6-31G(d) as previously done in Ref.[7]. Comparison between the static approach and more-advanced sampling schemes and approaches has been discussed elsewhere by some of us.[26]

The first important observation is that there are several high and low frequency modes are active upon excitation (see Table S1). High frequency modes involve mainly C=C stretching modes and are responsible for the main vibronic progression, while delocalized low frequency modes (e.g., that around 234 cm^{-1}) broaden the main vibronic peaks. A second clear point is that CAM-B3LYP tends to overestimate the vibronic coupling, that is the 0-1 transition is too intense compared to experiment. This overestimation is corrected in this work by scaling the electron-vibration coupling, as explained below.

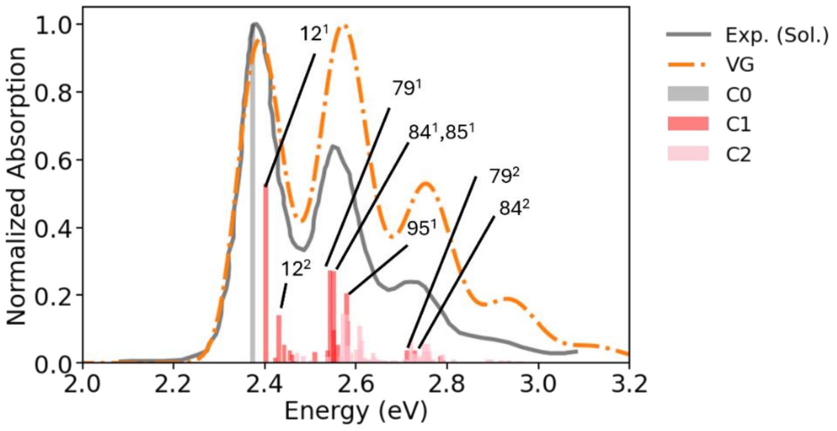

Figure S3: Comparison of computed and experimental spectra at room temperature using the Vertical Gradient approach. The computed VG spectrum was determined including all normal coordinates and shifted by -0.54eV to match the main experimental peak and it was broadened with a Gaussian with HWHM= 0.05 eV. The main stick bands of the computed VG spectrum are assigned with labels p^x, where p is the normal mode and x the number of quanta. Different colours distinguish stick bands to final states belonging to class C_n, where "*n*" is the number of simultaneously excited normal modes in the excited state. Experimental absorption spectrum was reproduced from Ref. 26, Copyright 2022 American Chemical Society.

As discussed in the main text, we are interested in investigating aggregates in condensed phase, thus a selection of the most important vibrations is necessary to make the problem computationally feasible. To this end, we have outlined an efficient Hierarchical effective mode selection (HEMS) and we adopted the effective mode reported in Table S1 to run QD wavepacket propagation on the excited S1 state of the isolated PDI molecule. Notably, in absence of non-adiabatic couplings between PESs, in principle QD simulations should yield the same results as demonstrated in other works [3]. In Fig. S4 we show that a fully converged spectra in agreement with the VG approach can be recovered with only a few blocks of the hierarchy. Note that both VG and QD simulations overestimate the vibronic progression with respect to experiment as TDDFT yields too large gradients. Thus, to correct for this electronic structure shortcomings we divided the gradient by a factor of 1.3, which allowed us to recover an almost perfect agreement with the experiment in terms of line-shape and peaks hight. We

note that, unless stated otherwise, the same scaling is applied to the gradients of the exciton, cation, and anion in all simulations of optical properties and excited-state dynamics of PDI aggregates presented in this work.

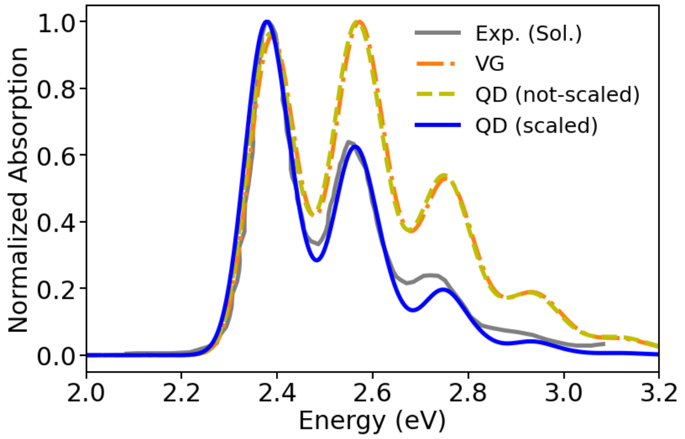

Figure S4: Absorption spectrum for the single monomer computed with VG and quantum dynamics (QD) with a fully converged numbers of hierarchically selected modes. Dashed yellow line represent the spectrum with unscaled gradient as obtained from TDDFT. While solid blue line represents the spectrum in which the gradients of the hierarchically selected modes are divided by 1.3. Experimental absorption spectrum was reproduced from Ref. 26, Copyright 2022 American Chemical Society.

Section S3.2: Normal modes analysis and hierarchical selection of modes

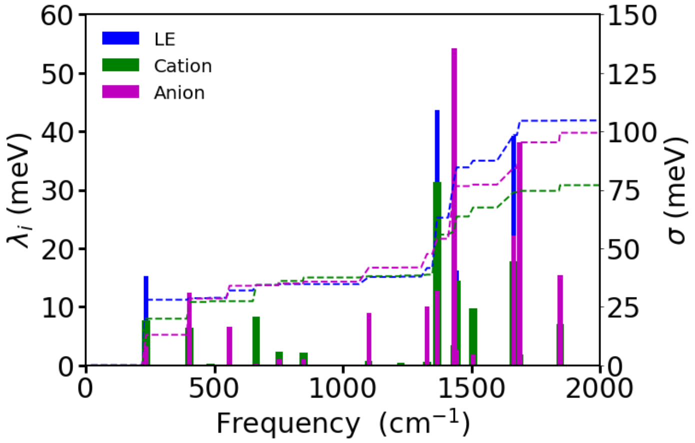

Figure S5: Relaxation energies of the different modes when going from the neutral to the excited state geometry, namely (a) anionic, (b) cationic and (c) excitonic state. On the second axis is the cumulated variance.

Table S1: Normal modes and related dimensionless displacement g_k^α, unscaled electron-vibrational coupling, $\lambda_k^{\alpha,LE}$, and relaxation energies, λ_α^{rel}, for the five most strongly coupled modes to the excitation.

**exciton**				
normal modes	ω [cm-1]	g_k^α	$\lambda_k^{\alpha,LE}$ (eV)	λ_α^{rel}(eV)
12	234.3	-1.023	-0.030	0.015
20	403.2	0.169	0.008	0.001

79	1367.0	-0.717	-0.122	0.044
84	1433.1	-0.714	-0.127	0.045
95	1664.3	-0.617	-0.127	0.039
Total Lambda (eV)				0.212
Hier. 1	1477.83	1.464	0.268	

Table S2: Normal modes and related dimensionless displacement g_k^α, unscaled electron-vibrational coupling, $\lambda_k^{\alpha,LE}$, and relaxation energies, λ_α^{rel}, for the five most strongly coupled modes to the cationic state.

**cation**				
normal modes	ω [cm-1]	g_k^α	$\lambda_k^{\alpha,LE}$	λ_α^{rel}
12	234.34	-0.727	-0.021	0.008
20	403.22	-0.509	-0.025	0.006
79	1367.04	-0.608	-0.103	0.031
84	1433.13	-0.197	-0.035	0.003
95	1664.27	-0.727	-0.150	0.055
Total Lambda (eV)				0.115
Hier. 1	1477.83	0.847	0.155	
Hier. 2	1470.58	0.602	0.110	

Table S3: Normal modes and related dimensionless displacement g_k^α, unscaled electron-vibrational coupling, $\lambda_k^{\alpha,LE}$, and relaxation energies, λ_α^{rel}, for the five most strongly coupled modes to the anionic state.

**Anion**				
normal modes	ω [cm-1]	g_k^α	$\lambda_k^{\alpha,LE}$	λ_α^{rel}
12	234.3	-0.477	-0.014	0.003
20	403.2	0.705	0.035	0.012
79	1367.0	-0.388	-0.066	0.013
84	1433.1	-0.781	-0.139	0.054
95	1664.3	-0.464	-0.096	0.022
Total Lambda (eV)				0.191
Hier. 1	1477.83	1.238	0.227	
Hier. 2	1470.58	-0.648	-0.118	
Hier. 3	1342.36	0.164	0.0272	

Section S3.3: Hamiltonian Parameters

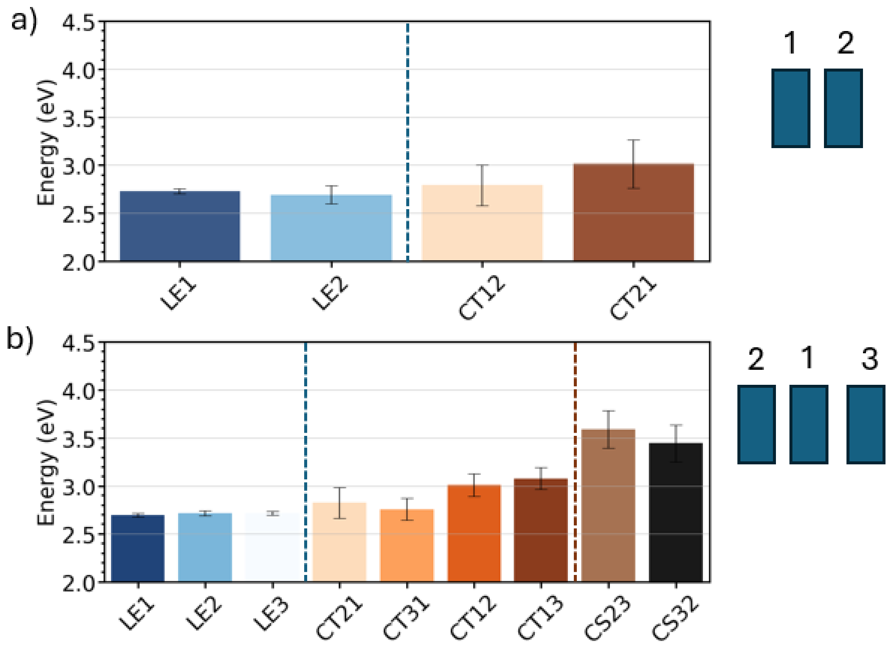

Figure S6: Site energy fluctuations for the different systems: a) dimer and b) trimer. The order of the molecules is reported in the inset on the right.

Table S4: Average couplings ($V = \langle|H_{kl}|\rangle$) and related standard deviations ($\sigma = \sqrt{\langle|H_{kl}|^2\rangle - \langle|H_{kl}|\rangle^2}$) the tetramer Hamiltonian.

	Dist. (Ang)	Couplings (meV)	σ (meV)	% fluctuation
V	3.74	93.5	15.5	16.6
	7.63	18.0	10.4	58.0
	10.80	6.8	4.4	63.9
D_h	3.74	71.6	45.4	63.4
	7.63	8.1	6.2	76.9
	10.80	0.9	0.8	86.1
D_e	3.74	32.5	18.3	56.4
	7.63	4.3	3.0	69.0
	10.80	0.5	0.5	93.6

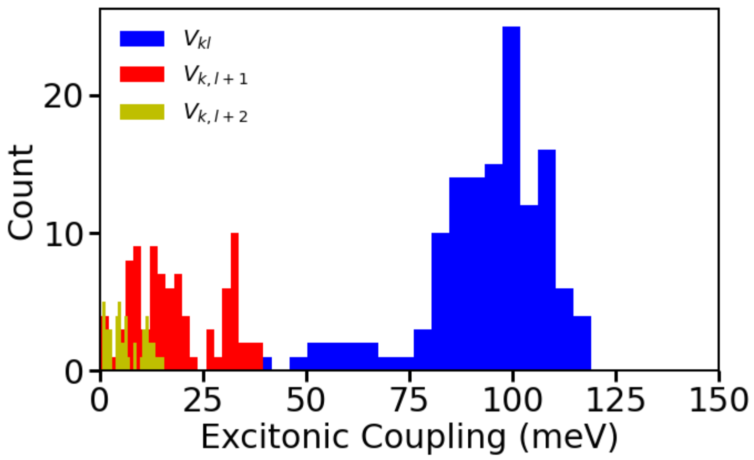

Figure S7: Histograms of the excitonic couplings V_{kl} computed for the tetramer system. V_{kl} is computed for nearest neighbour molecules (blue), separated by 1 molecule (red) and separated by 2 molecules (yellow)

Section S3.4: Geometric Analysis and Correlation with Electronic Hamiltonian parameters in the tetramer

The structural evolution of the PDI tetramer was analyzed using a set of supramolecular descriptors that quantify the relative positions and orientations of the individual chromophores. Each PDI unit was assigned a molecular reference frame defined by the long, short, and normal axes of its aromatic scaffold, denoted as $\hat{u}_{k,\parallel}$, $\hat{u}_{k,s'}$, and $\hat{u}_{k,\perp}$, respectively. Following the scheme developed by Martínez and co-workers[34] and later extended by Cantina et al.[35], the relative geometry between two PDI units (k, l) was characterized by eight descriptors: four translational parameters (ρ, r_π, r_l, and r_s) and four angular parameters (α, $\gamma1$, $\gamma2$, and δ) (see Table S5 for a description). The translational descriptors represent the center-of-mass distance and the relative displacements along the stacking, longitudinal, and side directions, while the angular descriptors describe spinning, rolling, tumbling, and dephasing motions between the molecular planes.[35]

To ensure a consistent treatment of equivalent configurations, all angular descriptors were considered with 180° periodicity, except for the spinning (yaw) angle α. For α, equivalent orientations were folded into the first quadrant instead of enforcing a 180° periodicity. In this way, mirror-related geometries—for instance, α = 30° and 150°—were mapped to the same effective value, removing artificial discontinuities between reflected configurations and providing a smooth representation of equivalent molecular orientations.

Electronic Hamiltonians were computed for each snapshot extracted from the classical MD trajectory. After diabatization (as discussed in the main text), we obtained local excitations (LE1–4), charge-transfer (CT), and charge-separated (CS) diabatic states. To rationalize how the structural fluctuations affect these quantities, each Hamiltonian element was associated with the instantaneous two-body geometry of the corresponding chromophore pair. In this framework, each "body" represents an individual PDI unit, and descriptors were evaluated for all relevant pairs within the tetramer, not only for nearest neighbours (e.g., including 1–3 and 1–4 interactions). The resulting set of pairwise geometric descriptors, summarized in Table S5, was therefore linked to the Hamiltonian elements to quantify how specific relative orientations and displacements between PDI units influence the electronic couplings and state energies.

To identify which geometrical features most strongly influence the electronic couplings, correlations were computed between the structural descriptors and the Hamiltonian elements. The set of variables $\{X\}$ includes the translational descriptors associated with interchromophore distances (ρ, r_π, r_l, and r_s), while the set $\{c_\theta\}$ contains the angular descriptors derived from the cosine-transformed angles ($\alpha, \gamma 1, \gamma 2$, and δ). These quantities were evaluated for each PDI pair over the ensemble of geometries extracted from the MD trajectory. Correlations were then calculated between the absolute geometric descriptors and the electronic Hamiltonian elements, $\{X, c_\theta\}$ vs. the Hamiltonian elements.

Two statistical complementary measures were employed: Pearson correlation coefficients to quantify linear dependencies and Spearman rank coefficients to capture monotonic but nonlinear trends. The resulting correlations are shown in Figure S8. Large absolute correlation values ($|r| \gtrsim 0.5$) were interpreted as indicators of strong structure–electronic coupling correlation. Both correlation maps reveal a pronounced relationship between the distance parameters and the electronic interactions, as also illustrated in Fig. 2b of the main text. In contrast, the angular descriptors generally display weaker correlations, reflecting the broader configurational flexibility of the aggregate. Notably, the spinning (yaw) angle α and the dephasing angle δ show localized enhancements, with α influencing short-range coupling strength and δ more effectively capturing collective columnar motions within the stack. Overall, this analysis provides a direct and quantitative link between the instantaneous two-body geometry of the aggregate and the evolution of its electronic Hamiltonian along the MD trajectory, offering microscopic insight into how molecular motions modulate excitonic interactions in multichromophoric assemblies.

Table S5: Summary of geometric descriptors between two PDI units and their physical meaning. Refer to Ref.[35] for their graphical representation.

Descriptor	Type	Description
ρ	Distance	Center-of-mass separation
r_π	Distance	Separation between the aromatic π-planes
r_l	Distance	Displacement along molecular long axis
r_s	Distance	Displacement along molecular short axis
α	Angle	Spinning (yaw) rotation
γ_1	Angle	Rolling (pitch) rotation
γ_2	Angle	Tumbling (roll) rotation
δ	Angle	Dephasing relative to columnar axis

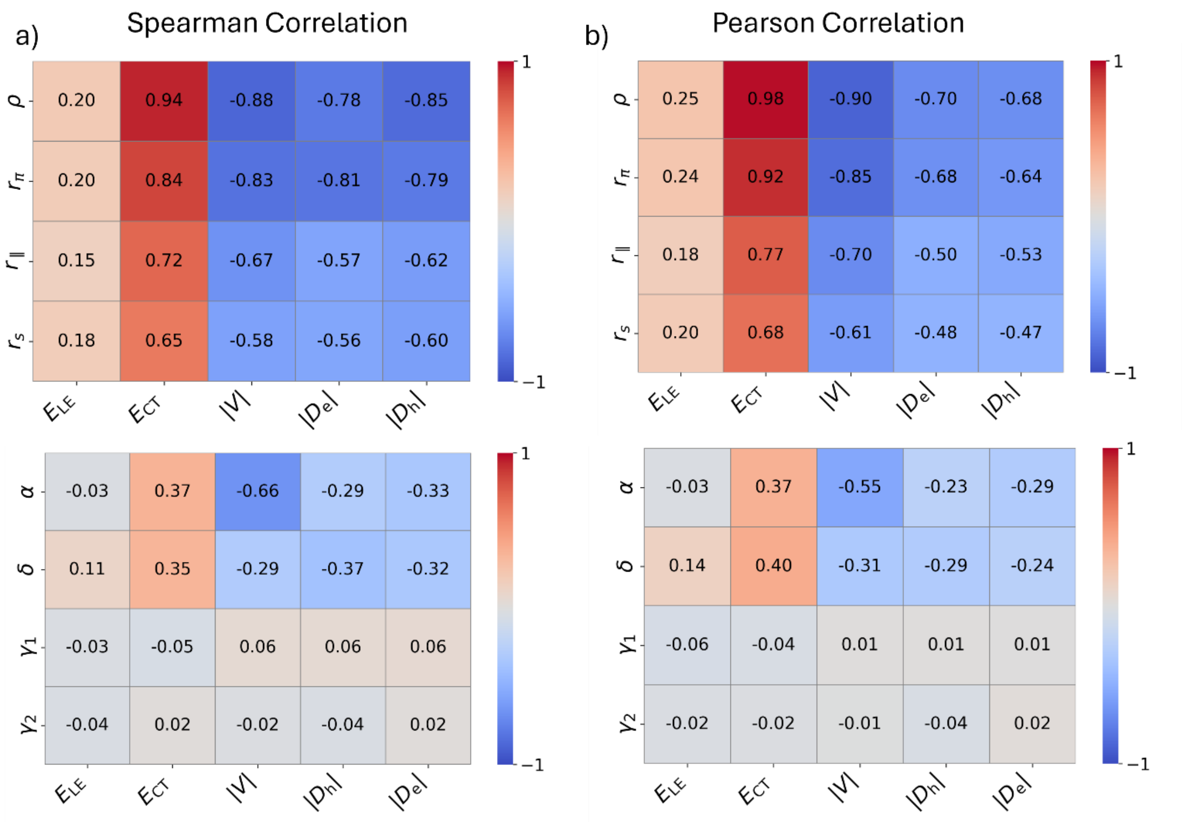

Figure S8: Spearman and Pearson correlations between parameters of the Hamiltonian and distance (top-panels) and angular (bottom-panels) descriptors. The boxed squares and their colors indicate the magnitude of the correlation between the energies and couplings shown on the x-axis and the geometric descriptors on the y-axis. Refer to Table S5 for a description of the geometric descriptors.

Section S3.5: Environmental effects on the Hamiltonian parameters

In Fig. S9, we performed diabatization excluding the explicit solvent molecules in the computation of the adiabatic states of the aggregate as well as in the monomer reference states needed for the diabaization procedure (see Section S2.3). The same figure as in the main text is plotted for a direct comparison.

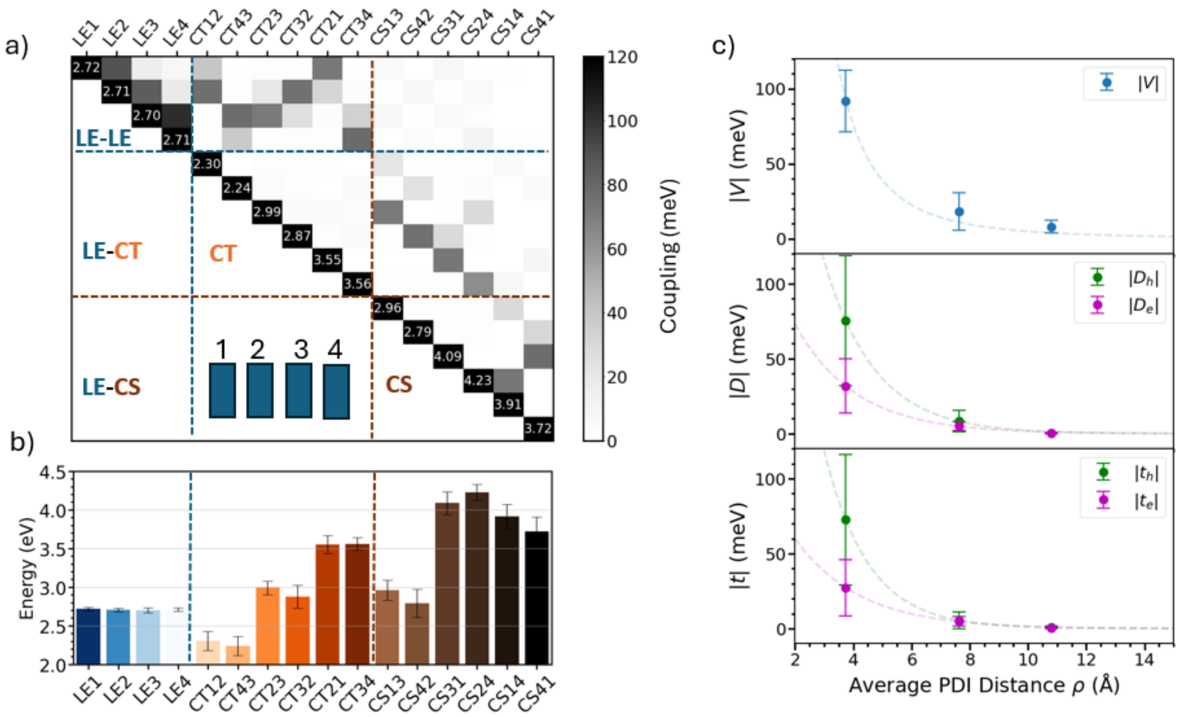

Figure S9: This figure shows the same panels as in Fig.2 of the main text but removing explicit water molecules from the diabatization performed as detailed in Section S2.3.

Section S3.6: Convergence with hierarchy blocks

In Figure S10 we show that 2 blocks (12 coordinates for a dimer, 18 for a trimer and 24 for a tetramer) already provide converged spectra for a Gaussian broadening with a half width at half-maximum (HWHM) of 0.032 eV and that including a third block results in very minor changes. Therefore, in most of the simulations two blocks were adopted to compute the spectra of all snapshots, unless stated otherwise.

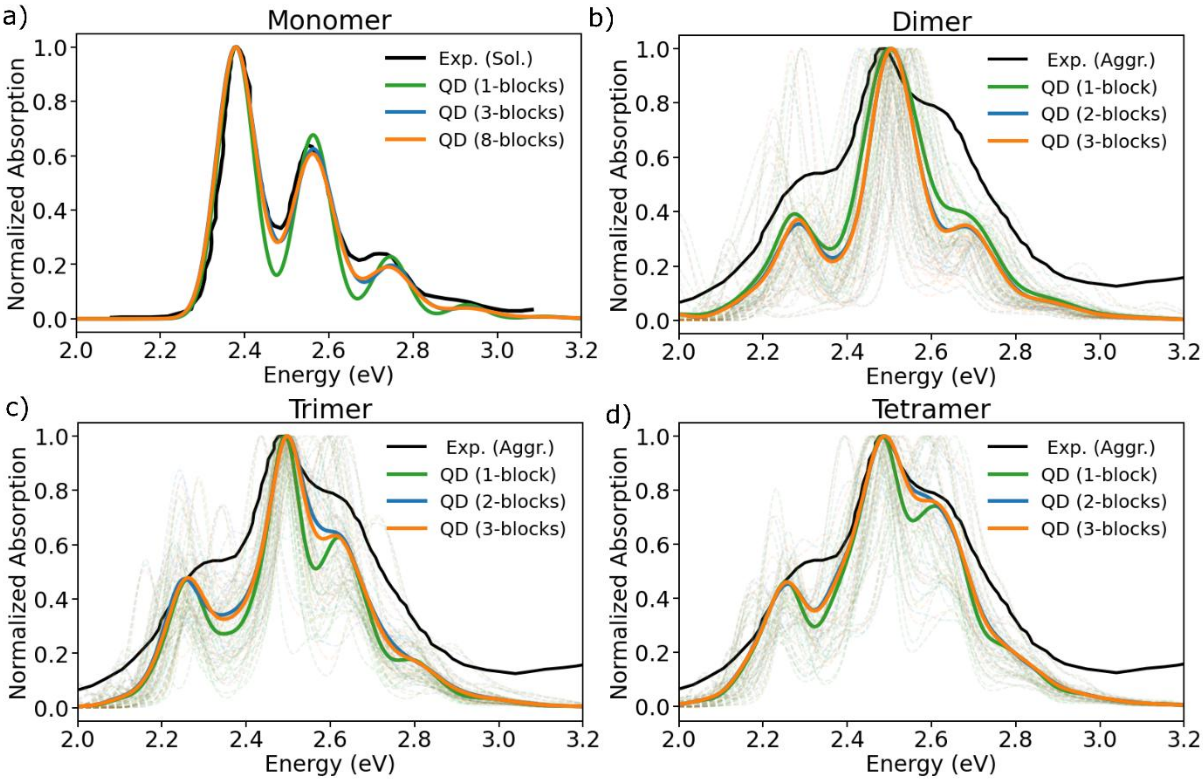

Figure S10: a) Absorption for the monomer in vacuo built for the optimized structure. Vertical energy taken from CAM-B3LYP in gas-phase (2.855 eV). The gas-phase spectrum was red Shift=-0.32 eV to match the experiment. A Gaussian with a HWHM of 0.036 eV was used for the monomer. Panels b), c), d) represent dimer, trimer and tetramer aggregate in water. Gradients scaled by factor 1.3, shift=-0.32 eV. A Gaussian of HWHM = 0.032 eV was used dimer, trimer and tetramer. The experimental absorption spectra were reproduced from Ref. 26, Copyright 2022 American Chemical Society.

Section S3.7: Convergence with number of snapshots

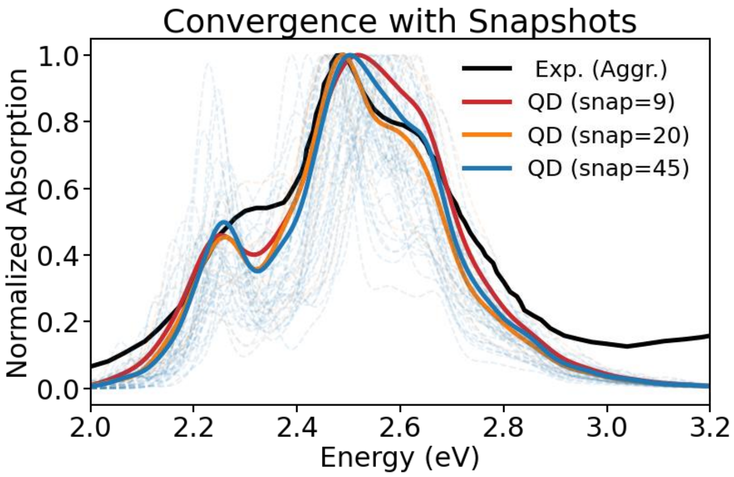

Figure S11: Convergence with number of snapshots for a tetramer aggregate used to create the average spectra. As the number of snapshots increases the phenomenological broadening used for the spectrum of each realization can be decreased. Thus, the HWHM goes from 0.037 eV for 9 snapshots to 0.031 eV for 20 snapshots, to 0.024 eV for 43 snapshots. The experimental absorption spectrum was reproduced from Ref. 26, Copyright 2022 American Chemical Society.

Section S3.8: Check the effect of constructing an averaged Hamiltonian

In Figure S12, we report the spectrum obtained by first computing the average of the parameters over MD snapshots and then performing QD with a converged number of blocks.

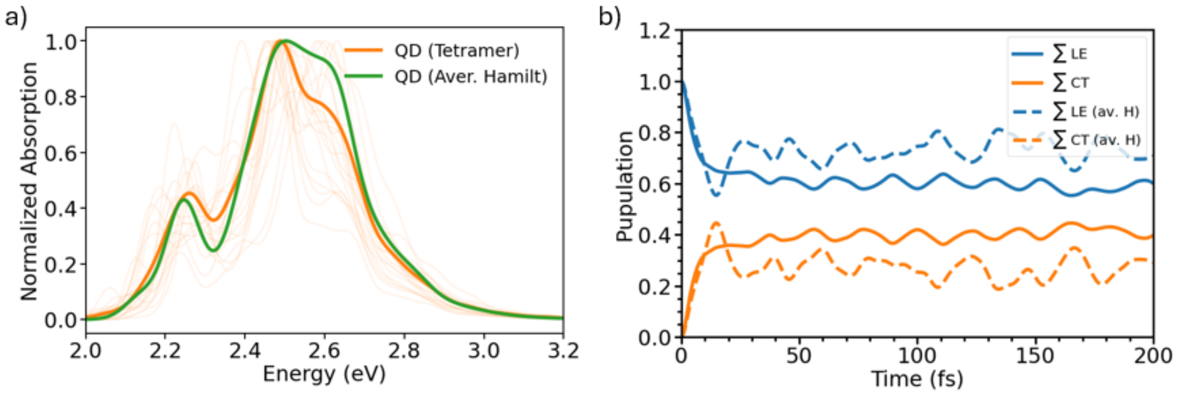

Figure S12: a) Average Hamiltonian was constructed by averaging the Hamiltonian matrix elements for all snapshots. The correct sign consistency of the couplings was ensured controlling the phase of the involved orbitals. b) Represent the evolution of the summed population of LE states and CT states average over all trajectories for the averaged Hamiltonian (solid lines) and the fluctuating one (dashed line).

Section S3.9: Comparison with and without CTs

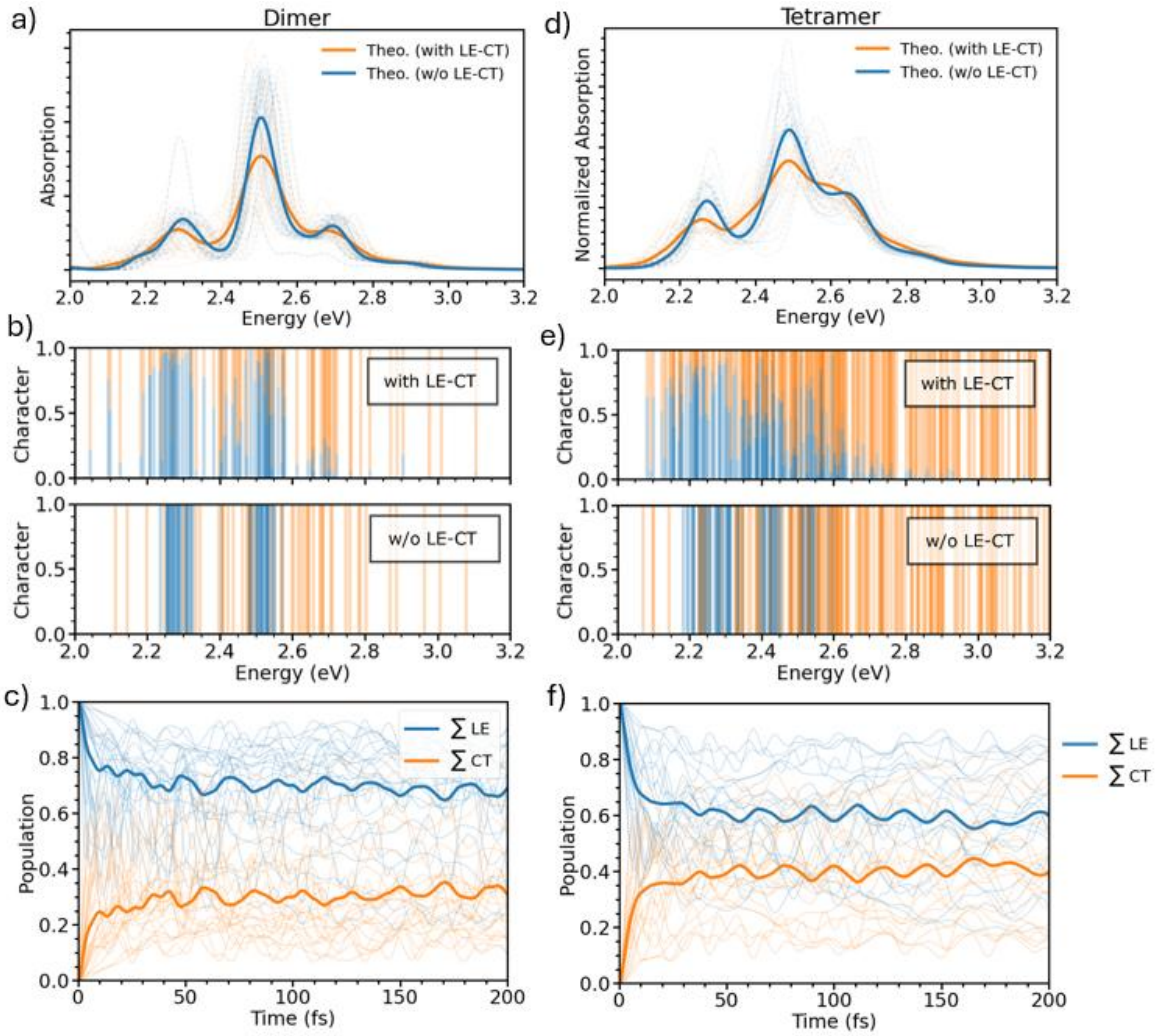

Figure S13: a), d) upper panels absorption spectra of a dimer and a tetramer, respectively, simulated including CT excitation (solid orange lines) or considering only LE states excluding their interaction with CT states (solid blue lines). The calculated spectra were shifted by 0.32 eV, and all transitions were convoluted with a Gaussian of 0.03 eV. Thin blue and orange lines represent single quantum dynamics (QD) trajectories calculated for each MD snapshot. b), e) Middle panels show the character (Eq. S30, S31) and density of the states for both dimer and tetramer the presence of LE-CT mixing, while the lower panel show the same but excluding LE-CT mixing. Panels c) and f) represent the evolution of the summed population of LE states and CT states over all trajectories. Individual trajectories are represented with thin-lines.

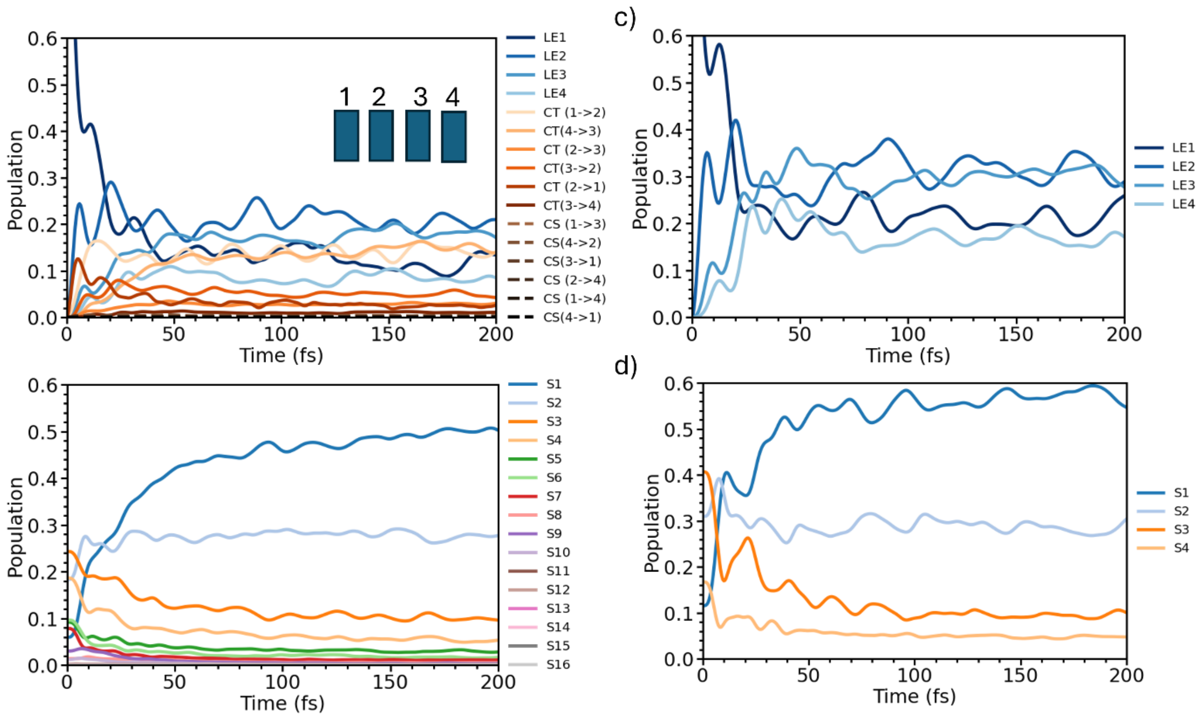

Figure S14: Population evolution tetramer with (left panes) and without (right panels) including CT states. Diabatic population are reported in panel a) and c), with and without including CT states, respectively. Pseudo-adiabatic population evolution with (panel b) and without (panel d) including CT states. The Pseudo-adiabatic population has been computed by rotating the diabatic states with the adiabatic-to-diabatic transformation matrix computed at the FC point (that is at time 0).

Section S3.10: Population evolution of dimer and trimer aggregates

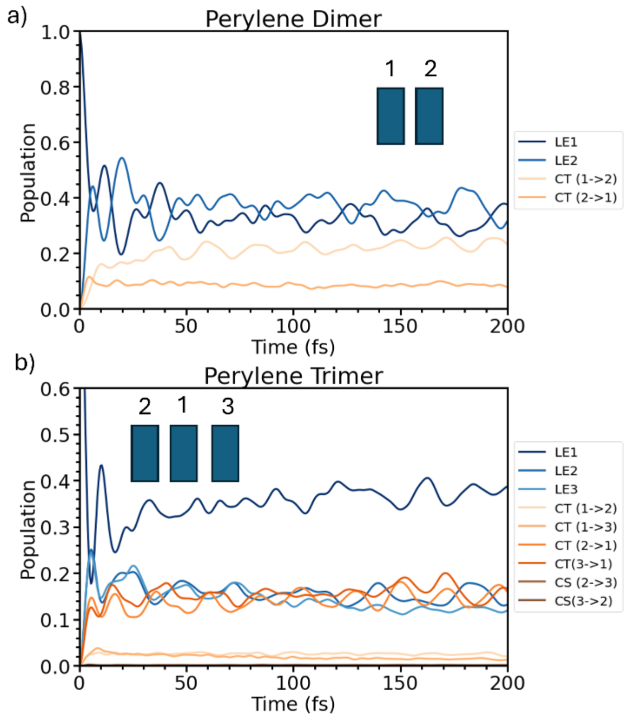

Figure S15: Population evolution of dimer (panel a) and trimer (panel b) using 3 blocks of the hierarchy.

Section S3.11: Tetramer: coherence and dynamics

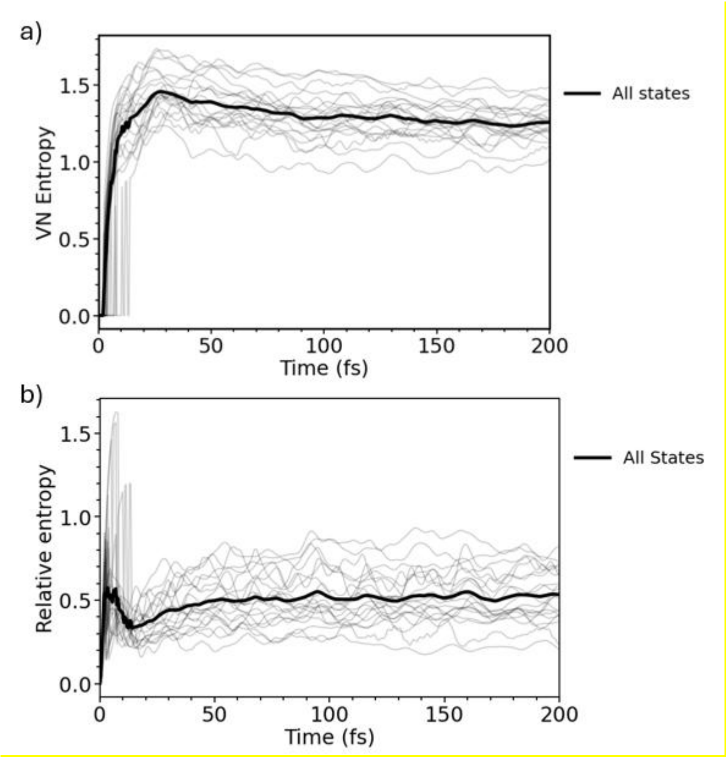

Figure S16: a) Von Neumann entropy as a function of time computed with Eq. S28 for the tetramer. Thin lines represent the quantity computed for each trajectory initiated from a different MD snapshot, while thick lines represent the average over all trajectories. b) Relative entropy as a function of time computed with Eq. S29 for the tetramer. Thin lines represent the quantity computed for each trajectory initiated from a different MD snapshot, while thick lines represent the average over all trajectories.

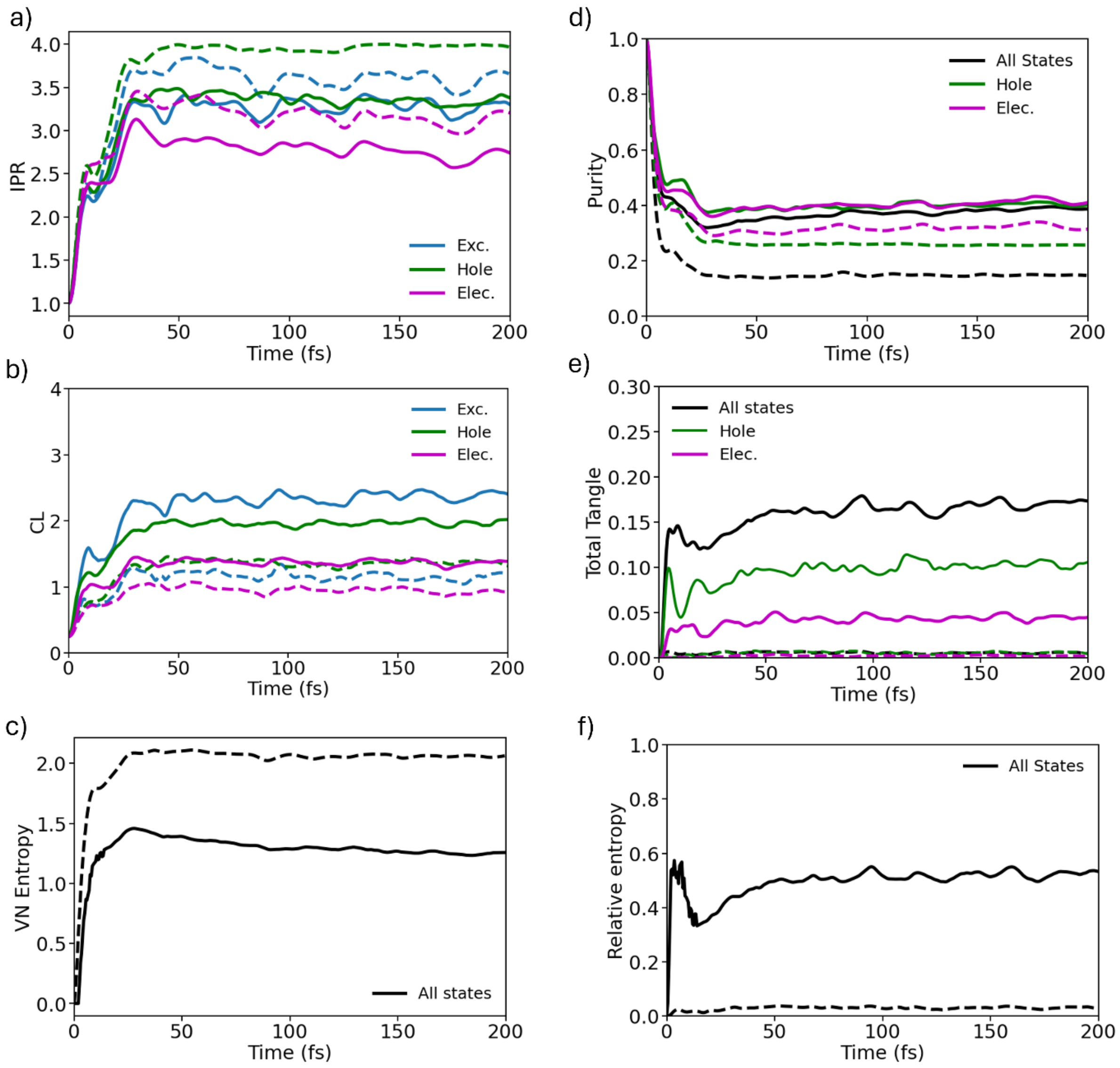

Figure S17: Different quantities used in other figures, computed for the tetramer using two approaches to averaging the density matrix. Solid lines indicate the results obtained by first computing the density matrix and thus each quantity for individual trajectories and then averaging over all trajectories. Dashed lines show the results obtained by first averaging the density matrix over all trajectories and then computing each quantity from this averaged density matrix.

Section S3.12: Tetramer coherence and dynamics with and without CT

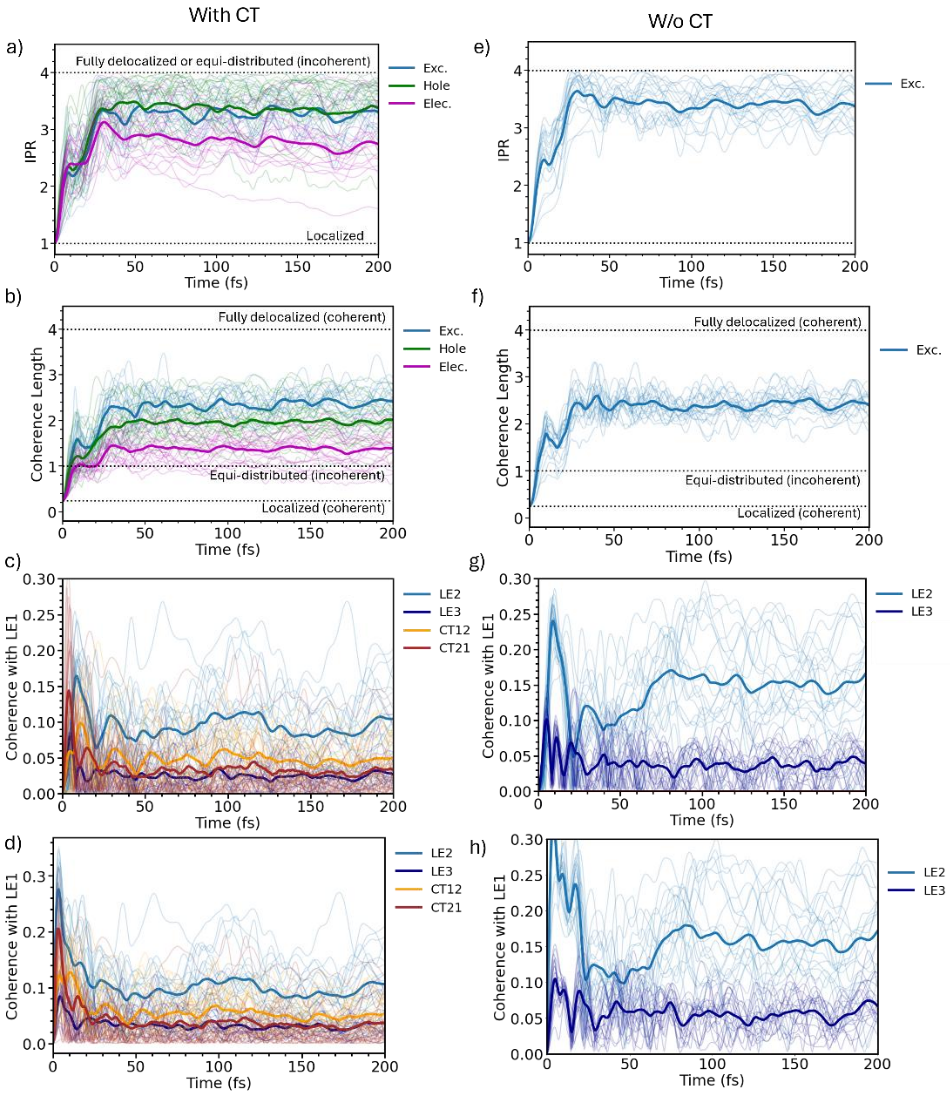

Figure S18: IPR, Coherence length, real part of the coherence and absolute coherence between the LE1 state and the indicated diabatic states with (panel a,b,c,d) respectively, and without (panel e,f,g, h) including CT states in the wave packet propagation.

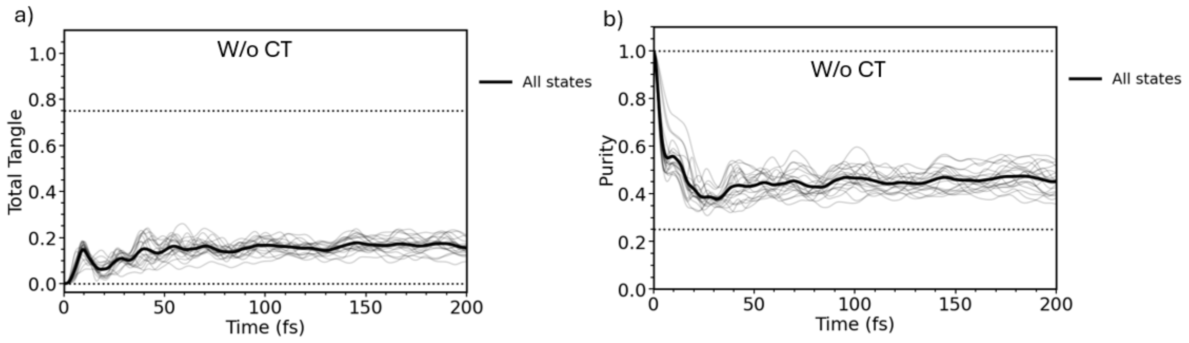

Figure S19: Total tangle (panel a) and Purity (panel b) for the trajectories where CT states are not included in the wavepacket dynamics. Note that the limits are different from those reported in Fig. 4 of the main text as the aggregate without CT reported here has only 4 states in total.

Section S3.13: Tetramer coherence and dynamics starting from bright adiabatic states

To test the robustness of our conclusions on the excited-state dynamics of the tetramer, we also considered an alternative wavefunction initialization in which the system is prepared in the brightest state accessible as if it was prepared by a broadband excitation. This state was identified using an adiabatic-to-diabatic transformation to rotate the electronic states and select the one with the largest squared transition dipole moment. The subsequent QD propagation was still performed in the diabatic basis. After an initial relaxation, interestingly the population dynamics become essentially independent of the chosen initial condition (See Fig. S21 and S14).

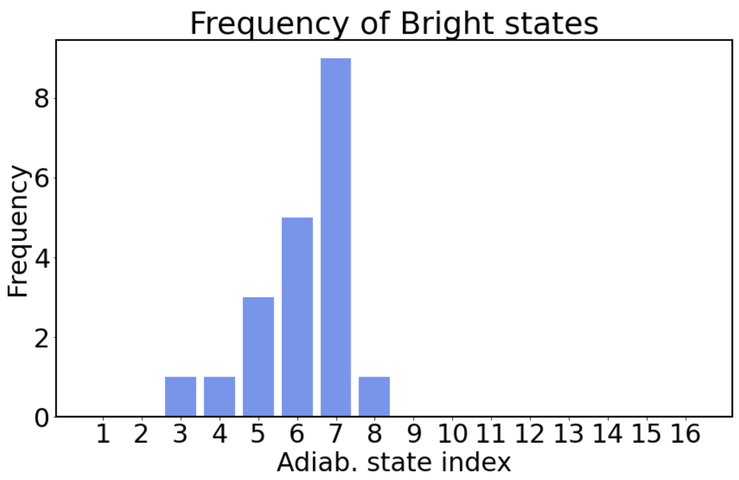

Figure S20: Histogram of the frequency (count) with which the index brightest state (i.e. largest squared module of the transition dipole) appears in each snapshot.

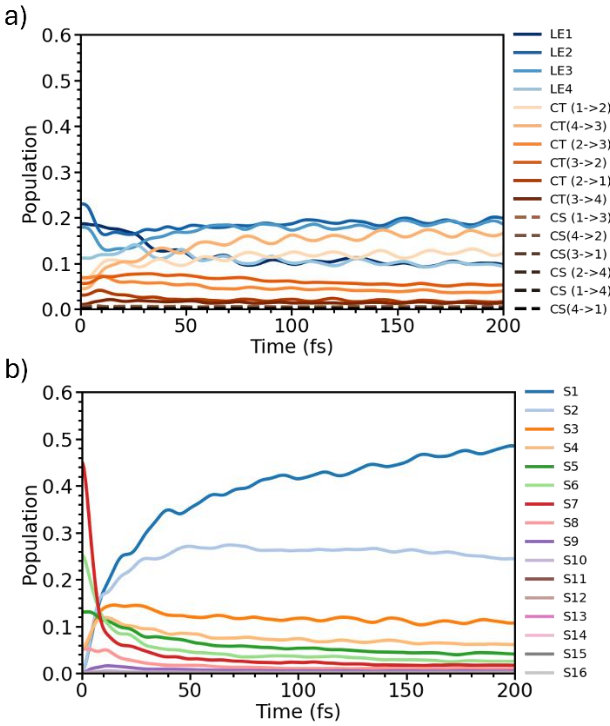

Figure S21: Population evolution starting from the brightest state of each snapshot. The evolution in the Diabatic basis is reported in panel (a) while the evolution in the pseudo-adiabatic basis defined at the FC point is reported in panel (b). Note that this figure should be compared with Fig S14a,b.

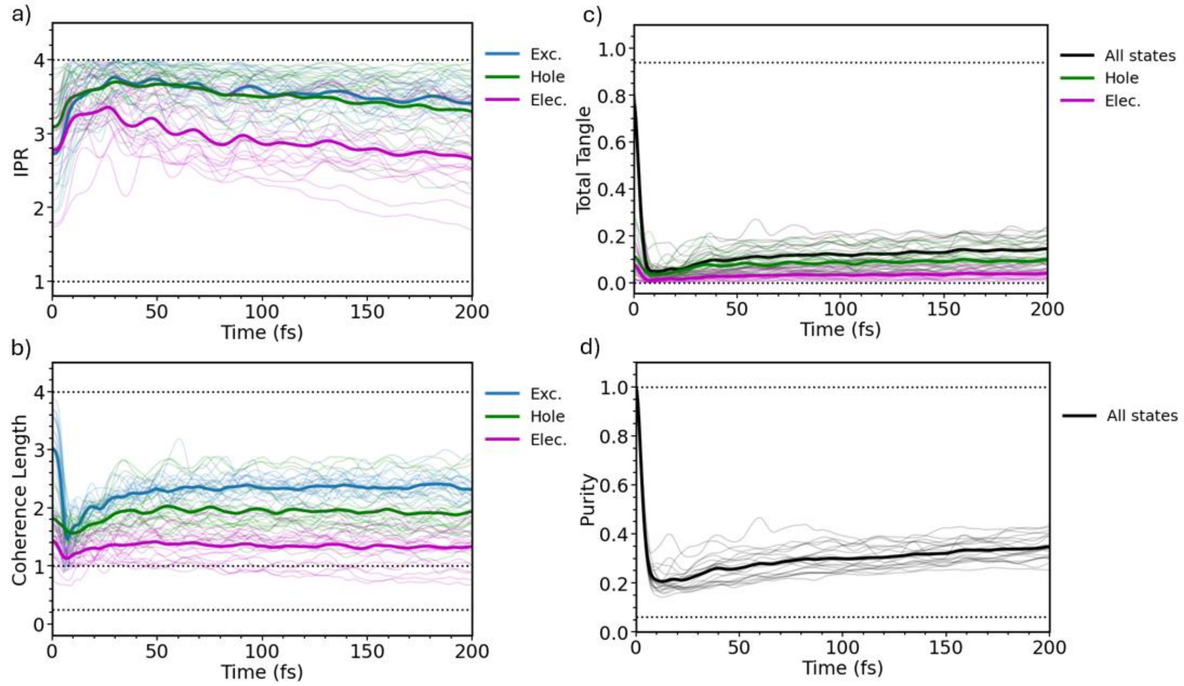

Figure S22: IPR, Coherence length, Total Tangle and purity computed for the QD trajectories started from the brightest adiabatic state as defined above.

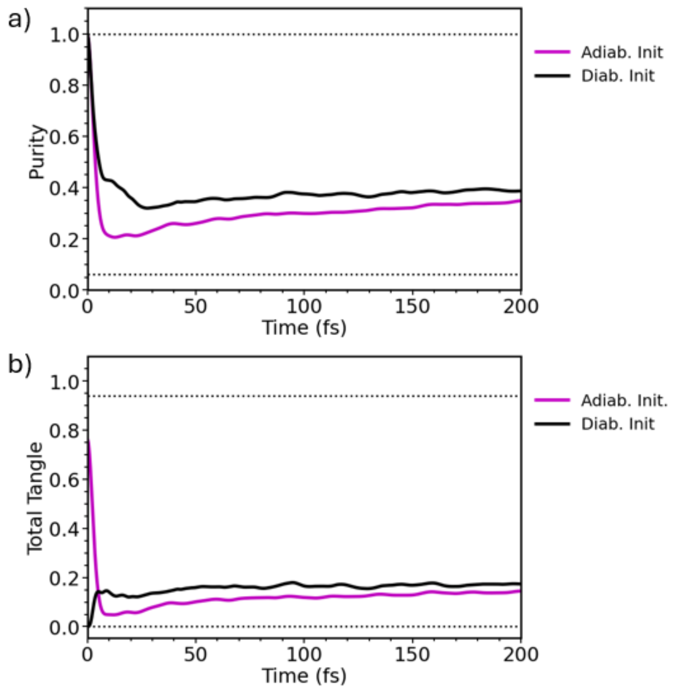

Figure S23: (a) Purity of the electronic density matrix, defined in Eq. S18 and (b) Total Tangle, defined in Eq. S27. Both these measures are performed for the physical system, in which the coupling to high-frequency vibrations provides the best agreement in the optical spectra with experiment (black line); the same system initialized from the brightest state of each snapshot (magenta line).

Section S3.14: Reduction electron-vibrational coupling

We report additional results regarding the analysis described in Section 2.6 of the main text.

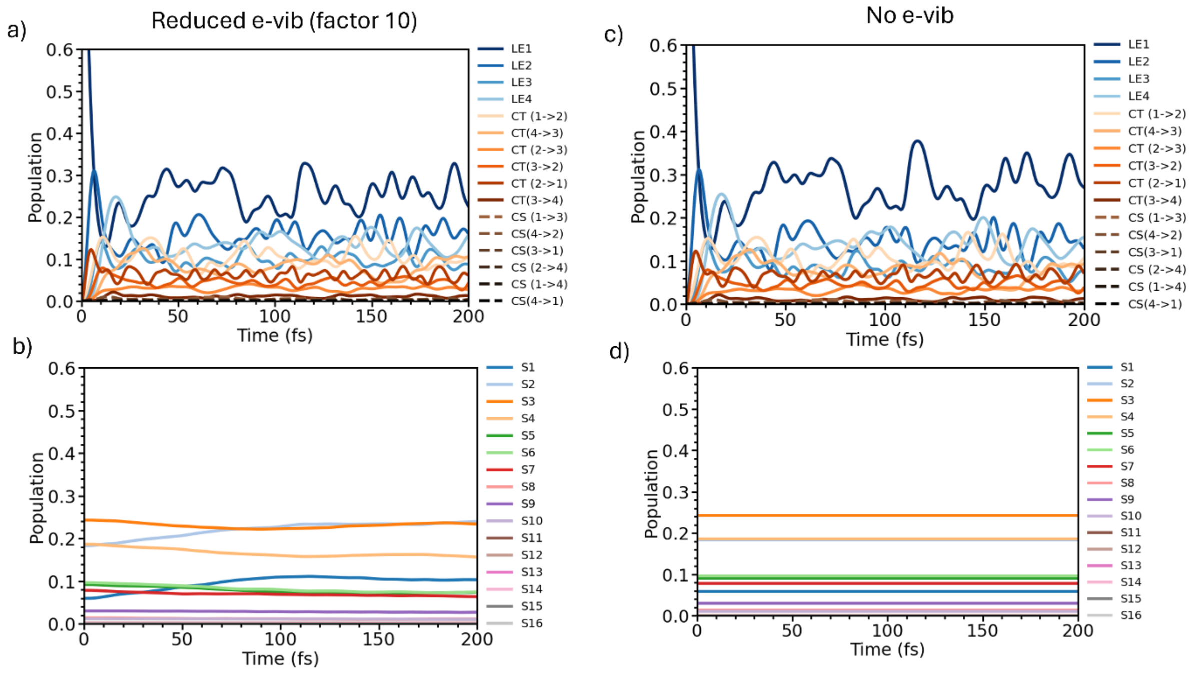

Figure S24: Population evolution tetramer reducing the electron-vibration couplings (i.e. the gradients, see Section S1) by factor 10 (left panes) and completely (right panels). Diabatic populations are reported in panel a) and c), respectively. Pseudo-adiabatic population evolutions are reported in panels b), d).

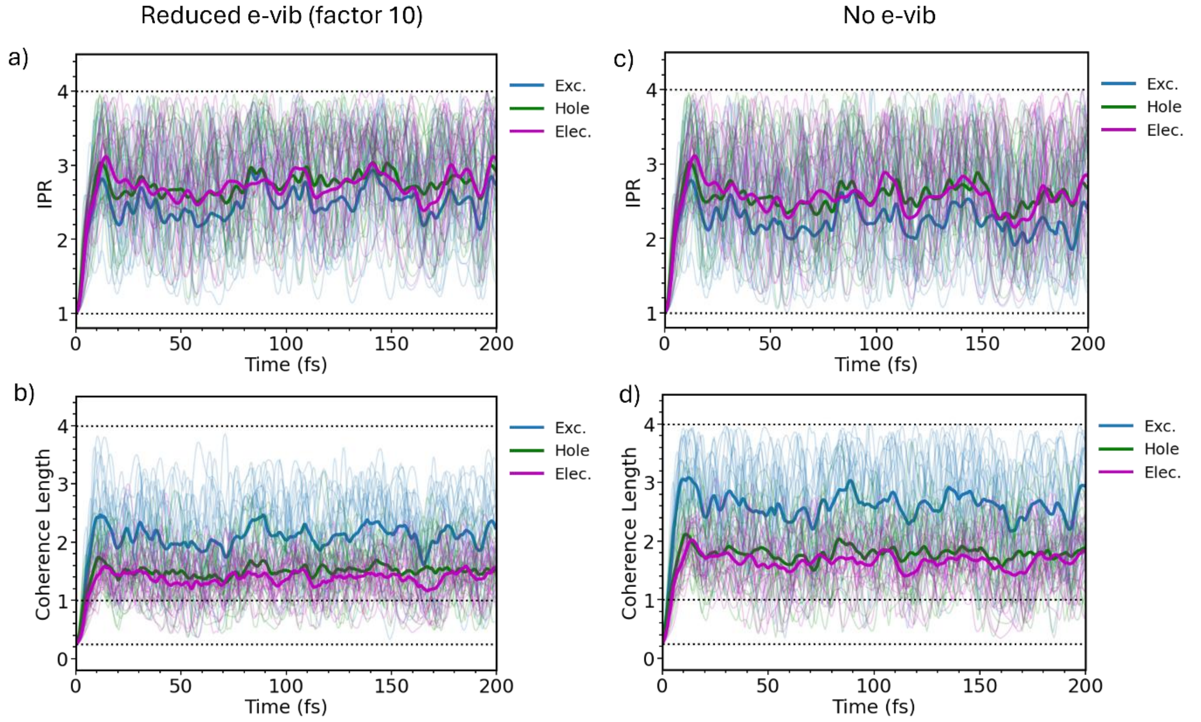

Figure S25: IPR, Coherence length reducing the electron-vibration couplings (i.e. the gradients, see Section S1) by factor 10 (left panes) and completely (right panels).

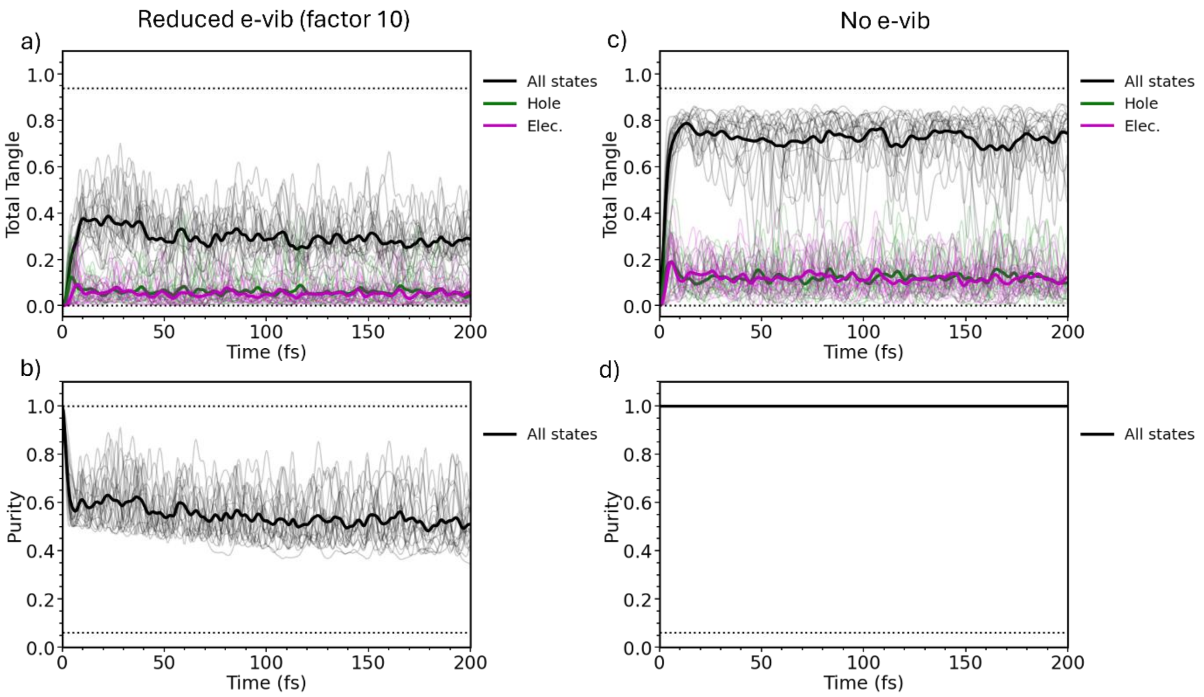

Figure S26: Total tangle and Purity reducing the electron-vibration couplings (i.e. the gradients, see Section S1) by factor 10 (left panes) and completely (right panels).

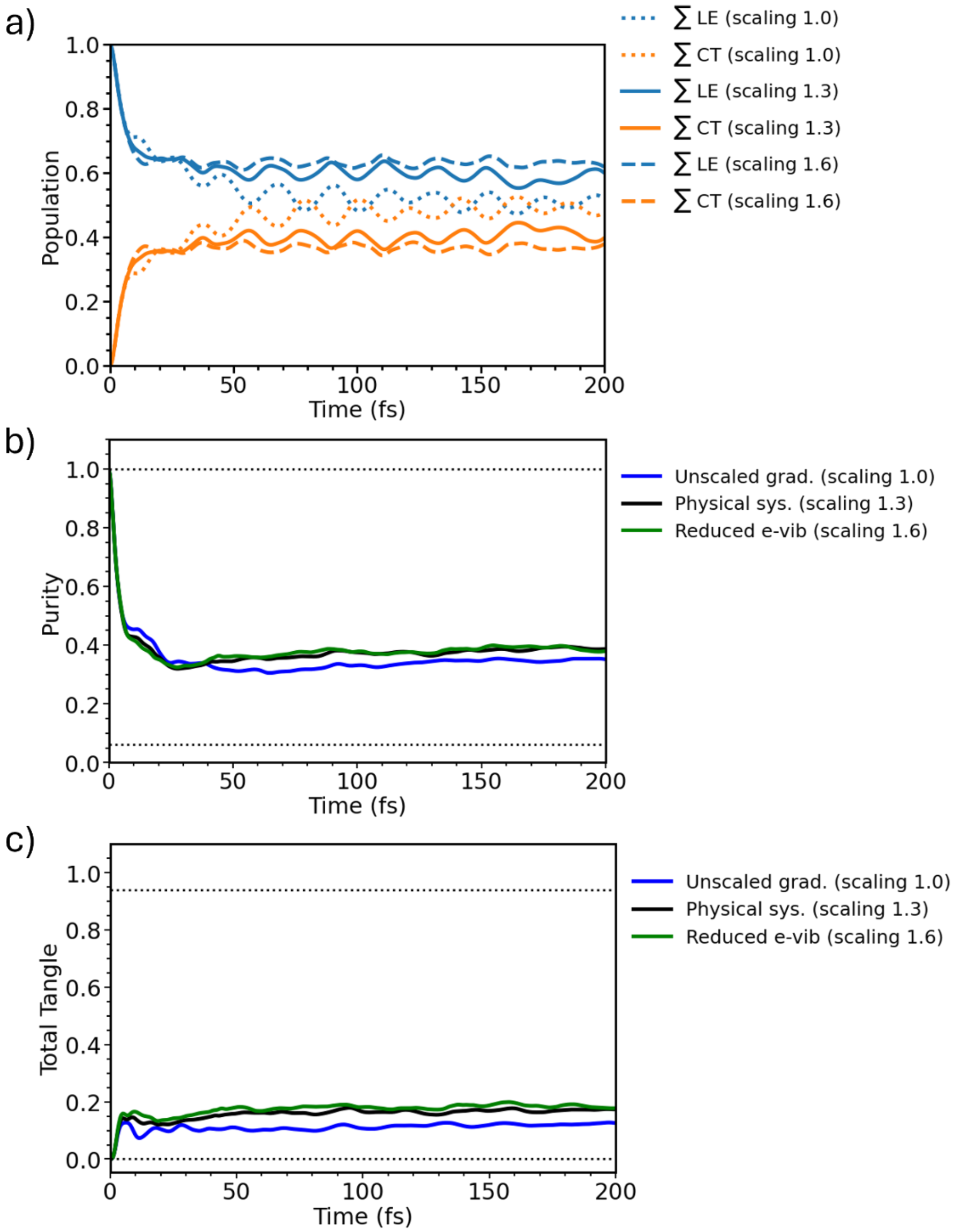

Figure S27: Sensitivity analysis to gradient scaling factor going from 1.0 to 1.6 on sum of LE and CT populations (panel a), Purity (panel b) and Total Tangle (panel c). For scaling factors 1.0 and 1.6 a reduced number of trajectories (20 in total) was run.

Section 3.15: Comparison with previous work

Here, we provide a comparison with previous work in Refs.[26] and Ref.[7]. We note that this comparison is performed using unscaled gradients as obtained directly from TDDFT. The scaling factor was introduced only in the present work to correct for the known overestimation of CAM-B3LYP functional as discussed in Section S3. 1.

We begin by reproducing the simulation shown in Fig. 9a of Ref.[26] (Segalina et al., 2020 in magenta) in Fig. S28a and comparing it with the monomer spectrum obtained in this work (see Fig. 1c). The slight differences in peak intensities arise from the fact that, in the present work, the simulations are performed using the VG approach. This choice reflects the fact that VG corresponds to the limiting case to which the ad-MD|LVC method reduces in the absence of interstate couplings.

In Ref.[26], the primary objective was to achieve the most accurate possible description of the monomer, and the vibronic-Hessian (VH) approach was therefore adopted, explicitly accounting for Duschinsky mixing effects. In the present work, instead, the monomer is used solely to validate the gradients entering Eq. S11, i.e., the vibronic part of the Hamiltonian. The methodological choices in the two studies thus reflect their different scientific goals, while the resulting physical pictures remain fully consistent.

Next, we compared the dimer simulation shown in Fig. 6 of Ref.[7] (Segalina et al., 2022 in magenta), reproduced in Fig. S28b, with the dimer simulation presented in the current work. When performed using unscaled gradients, the two simulations are equivalent. The minor differences observed arise from the different sampling protocols: 100 snapshots were used in Ref.[7], whereas only 25 snapshots are employed in the present study, as the dimer is included here only to illustrate that larger aggregates are required to quantitatively reproduce the experimental observations.

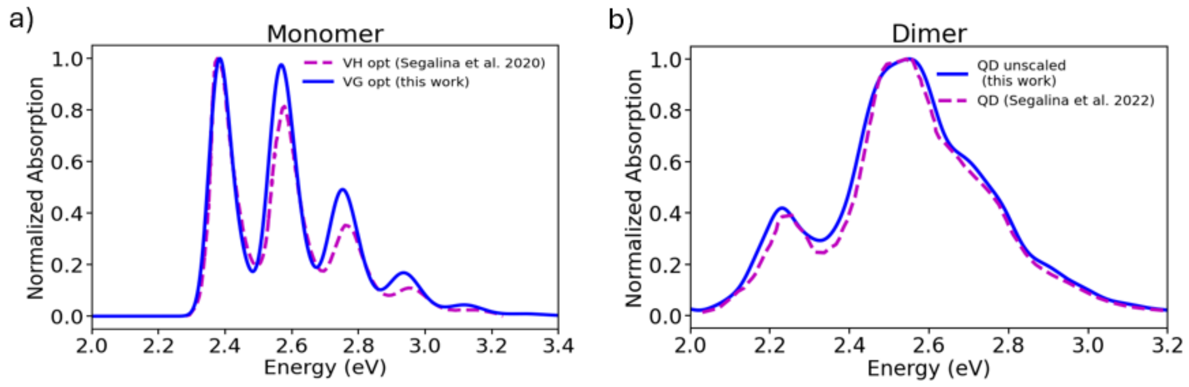

Figure S28: Comparison results in this work and Ref. [26] for the PDI monomer (panel a) and Ref.[7] (panel b) for the dimer in solution. In panels a) and b), the *x,y* data reproduced in magenta were taken from Ref. 26 and Ref. 7, respectively. Copyright 2020 and 2022 American Chemical Society, respectively. Note that the comparison must be done for unscaled couplings. For the monomer the applied broadening is HWHM=0.032 eV (adjusted for better comparison with Ref. 26). For the dimer the applied broadening is HWHM=0.032 eV, while in Ref. 26 HWHM=0.03 eV was used.

References

(1) Cerezo, J.; Santoro, F. FCclasses3 : Vibrationally-resolved Spectra Simulated at the Edge of the Harmonic Approximation. *J. Comput. Chem.* **2023**, *44* (4), 626–643. https://doi.org/10.1002/jcc.27027.

(2) Aarabi, M.; Aranda, D.; Gholami, S.; Meena, S. K.; Lerouge, F.; Bretonniere, Y.; Gürol, I.; Baldeck, P.; Parola, S.; Dumoulin, F.; Cerezo, J.; Garavelli, M.; Santoro, F.; Rivalta, I. Quantum-Classical Protocol for Efficient Characterization of Absorption Lineshape and Fluorescence Quenching upon Aggregation: The Case of Zinc Phthalocyanine Dyes. *J. Chem. Theory Comput.* **2023**. https://doi.org/10.1021/acs.jctc.3c00446.

(3) Aranda, D.; Santoro, F. Vibronic Spectra of π-Conjugated Systems with a Multitude of Coupled States: A Protocol Based on Linear Vibronic Coupling Models and Quantum Dynamics Tested on Hexahelicene. *J. Chem. Theory Comput.* **2021**, *17* (3), 1691–1700. https://doi.org/10.1021/acs.jctc.1c00022.

(4) Avila Ferrer, F. J.; Santoro, F. Comparison of Vertical and Adiabatic Harmonic Approaches for the Calculation of the Vibrational Structure of Electronic Spectra. *Phys. Chem. Chem. Phys.* **2012**, *14* (39), 13549. https://doi.org/10.1039/c2cp41169e.

(5) Worth, G. A. Quantics: A General Purpose Package for Quantum Molecular Dynamics Simulations. *Comput. Phys. Commun.* **2020**, *248*, 107040. https://doi.org/10.1016/j.cpc.2019.107040.

(6) Worth, G. A.; Meyer, H. D.; Köppel, H.; Cederbaum, L. S.; Burghardt, I. *Using the MCTDH Wavepacket Propagation Method to Describe Multimode Non-Adiabatic Dynamics*; 2008; Vol. 27. https://doi.org/10.1080/01442350802137656.

(7) Segalina, A.; Aranda, D.; Green, J. A.; Cristino, V.; Caramori, S.; Prampolini, G.; Pastore, M.; Santoro, F. How the Interplay among Conformational Disorder, Solvation, Local, and Charge-Transfer Excitations Affects the Absorption Spectrum and Photoinduced Dynamics of Perylene Diimide Dimers: A Molecular Dynamics/Quantum Vibronic Approach. *J. Chem. Theory Comput.* **2022**, *18* (6), 3718–3736. https://doi.org/10.1021/acs.jctc.2c00063.

(8) Giannini, S.; Sowood, D. J. C.; Cerdá, J.; Frederix, S.; Grüne, J.; Londi, G.; Marsh, T.; Ghosh, P.; Duchemin, I.; Greenham, N. C.; Vandewal, K.; D'Avino, G.; Gillett, A. J.; Beljonne, D. On the Role of Charge Transfer Excitations in Non-Fullerene Acceptors for Organic Photovoltaics. *Mater. Today* **2024**, *80* (xx), 308–326. https://doi.org/10.1016/j.mattod.2024.09.009.

(9) Giannini, S.; Cerdá, J.; Prampolini, G.; Santoro, F.; Beljonne, D. Dissecting the Nature and Dynamics of Electronic Excitations in a Solid-State Aggregate of a Representative Non-Fullerene Acceptor. *J. Mater. Chem. C* **2024**, *12* (27), 10009–10028. https://doi.org/10.1039/D4TC01716A.

(10) Giannini, S.; Carof, A.; Ellis, M.; Ziogos, O. G.; Blumberger, J. Chapter 6. From Atomic Orbitals to Nano-Scale Charge Transport with Mixed Quantum/Classical Non-Adiabatic Dynamics: Method, Implementation and Application. In *Multiscale Dynamics Simulations: Nano and Nano-bio Systems in Complex Environments, Royal Society of Chemistry. eds Dennis R. Salahub; Dongqing Wei.*; 2021; pp 172–202. https://doi.org/10.1039/9781839164668-00172.

(11) Li, X.; Parrish, R. M.; Liu, F.; Kokkila Schumacher, S. I. L.; Martínez, T. J. An Ab Initio Exciton Model Including Charge-Transfer Excited States. *J. Chem. Theory Comput.* **2017**, *13* (8), 3493–3504. https://doi.org/,.

(12) Troisi, A. Charge Transport in High Mobility Molecular Semiconductors: Classical Models and

New Theories. *Chem. Soc. Rev.* **2011**, *40* (5), 2347. https://doi.org/10.1039/c0cs00198h.

(13) Hestand, N. J.; Spano, F. C. Interference between Coulombic and CT-Mediated Couplings in Molecular Aggregates: H- to J-Aggregate Transformation in Perylene-Based π-Stacks. *J. Chem. Phys.* **2015**, *143* (24). https://doi.org/10.1063/1.4938012.

(14) Hestand, N. J.; Spano, F. C. Expanded Theory of H- and J-Molecular Aggregates: The Effects of Vibronic Coupling and Intermolecular Charge Transfer. *Chem. Rev.* **2018**, *118* (15), 7069–7163. https://doi.org/10.1021/acs.chemrev.7b00581.

(15) Picconi, D.; Lami, A.; Santoro, F. Hierarchical Transformation of Hamiltonians with Linear and Quadratic Couplings for Nonadiabatic Quantum Dynamics: Application to the Ππ*/ n Π* Internal Conversion in Thymine. *J. Chem. Phys.* **2012**, *136* (24). https://doi.org/10.1063/1.4729049.

(16) Picconi, D. Dynamics of High-Dimensional Quantum Systems Coupled to a Harmonic Bath. General Theory and Implementation via Multiconfigurational Wave Packets and Truncated Hierarchical Equations for the Mean-Fields. *J. Chem. Phys.* **2024**, *161* (16). https://doi.org/10.1063/5.0233708.

(17) Gindensperger, E.; Burghardt, I.; Cederbaum, L. S. Short-Time Dynamics through Conical Intersections in Macrosystems. I. Theory: Effective-Mode Formulation. *J. Chem. Phys.* **2006**, *124* (14), 0–18. https://doi.org/10.1063/1.2183304.

(18) Gindensperger, E.; Burghardt, I.; Cederbaum, L. S. Short-Time Dynamics through Conical Intersections in Macrosystems. II. Applications. *J. Chem. Phys.* **2006**, *124* (14). https://doi.org/10.1063/1.2183305.

(19) Cederbaum, L. S.; Gindensperger, E.; Burghardt, I. Short-Time Dynamics through Conical Intersections in Macrosystems. *Phys. Rev. Lett.* **2005**, *94* (11), 1–4. https://doi.org/10.1103/PhysRevLett.94.113003.

(20) Scholes, G. D. Limits of Exciton Delocalization in Molecular Aggregates. *Faraday Discuss.* **2020**, *221*, 265–280. https://doi.org/10.1039/C9FD00064J.

(21) Smyth, C.; Fassioli, F.; Scholes, G. D. Measures and Implications of Electronic Coherence in Photosynthetic Light-Harvesting. *Philos. Trans. A. Math. Phys. Eng. Sci.* **2012**, *370* (1972), 3728–3749. https://doi.org/10.1098/rsta.2011.0420.

(22) Scholes, G. D.; Smyth, C. Perspective: Detecting and Measuring Exciton Delocalization in Photosynthetic Light Harvesting. *J. Chem. Phys.* **2014**, *140* (11). https://doi.org/10.1063/1.4869329.

(23) Fujita, T.; Atahan-Evrenk, S.; Sawaya, N. P. D.; Aspuru-Guzik, A. Coherent Dynamics of Mixed Frenkel and Charge-Transfer Excitons in Dinaphtho[2,3-b:2'3'-f]Thieno[3,2-b]-Thiophene Thin Films: The Importance of Hole Delocalization. *J. Phys. Chem. Lett.* **2016**, *7* (7), 1374–1380. https://doi.org/10.1021/acs.jpclett.6b00364.

(24) Sarovar, M.; Ishizaki, A.; Fleming, G. R.; Whaley, K. B. Quantum Entanglement in Photosynthetic Light-Harvesting Complexes. *Nat. Phys.* **2010**, *6* (6), 462–467. https://doi.org/10.1038/nphys1652.

(25) Cantina, M.; Padula, D.; Segalina, A.; Giannini, S.; Santoro, F.; Prampolini, G.; Pastore, M. Anticooperative Self-Assembly of Perylene Diimide Dyes in Water Unveiled by Advanced Molecular Dynamics Simulations. *Nanoscale* **2025**, *17* (40), 23626–23641. https://doi.org/10.1039/D5NR02723C.

(26) Segalina, A.; Cerezo, J.; Prampolini, G.; Santoro, F.; Pastore, M. Accounting for Vibronic Features through a Mixed Quantum-Classical Scheme: Structure, Dynamics, and Absorption Spectra of a Perylene Diimide Dye in Solution. *J. Chem. Theory Comput.* **2020**, *16* (11), 7061–7077. https://doi.org/10.1021/acs.jctc.0c00919.

(27) Green, J. A.; Yaghoubi Jouybari, M.; Asha, H.; Santoro, F.; Improta, R. Fragment Diabatization Linear Vibronic Coupling Model for Quantum Dynamics of Multichromophoric Systems: Population of the Charge-Transfer State in the Photoexcited Guanine–Cytosine Pair. *J. Chem. Theory Comput.* **2021**, *17* (8), 4660–4674. https://doi.org/10.1021/acs.jctc.1c00416.

(28) Frisch, M. J.; Trucks, G. W.; Schlegel, H. B.; Scuseria, G. E.; Robb, M. a.; Cheeseman, J. R.; Scalmani, G.; Barone, V.; Petersson, G. a.; Nakatsuji, H.; Li, X.; Caricato, M.; Marenich, a. V.; Bloino, J.; Janesko, B. G.; Gomperts, R.; Mennucci, B.; Hratchian, H. P.; Ortiz, J. V.; Izmaylov, a. F.; Sonnenberg, J. L.; Williams; Ding, F.; Lipparini, F.; Egidi, F.; Goings, J.; Peng, B.; Petrone, A.; Henderson, T.; Ranasinghe, D.; Zakrzewski, V. G.; Gao, J.; Rega, N.; Zheng, G.; Liang, W.; Hada, M.; Ehara, M.; Toyota, K.; Fukuda, R.; Hasegawa, J.; Ishida, M.; Nakajima, T.; Honda, Y.; Kitao, O.; Nakai, H.; Vreven, T.; Throssell, K.; Montgomery Jr., J. a.; Peralta, J. E.; Ogliaro, F.; Bearpark, M. J.; Heyd, J. J.; Brothers, E. N.; Kudin, K. N.; Staroverov, V. N.; Keith, T. a.; Kobayashi, R.; Normand, J.; Raghavachari, K.; Rendell, a. P.; Burant, J. C.; Iyengar, S. S.; Tomasi, J.; Cossi, M.; Millam, J. M.; Klene, M.; Adamo, C.; Cammi, R.; Ochterski, J. W.; Martin, R. L.; Morokuma, K.; Farkas, O.; Foresman, J. B.; Fox, D. J. G16_C01. 2016, p Gaussian 16, Revision C.01, Gaussian, Inc., Wallin.

(29) Meyer, H. D. Studying Molecular Quantum Dynamics with the Multiconfiguration Time-Dependent Hartree Method. *Wiley Interdiscip. Rev. Comput. Mol. Sci.* **2012**, *2* (2), 351–374. https://doi.org/10.1002/wcms.87.

(30) Beck, M. The Multiconfiguration Time-Dependent Hartree (MCTDH) Method: A Highly Efficient Algorithm for Propagating Wavepackets. *Phys. Rep.* **2000**, *324* (1), 1–105. https://doi.org/10.1016/S0370-1573(99)00047-2.

(31) Vendrell, O.; Meyer, H. D. Multilayer Multiconfiguration Time-Dependent Hartree Method: Implementation and Applications to a Henon-Heiles Hamiltonian and to Pyrazine. *J. Chem. Phys.* **2011**, *134* (4). https://doi.org/10.1063/1.3535541.

(32) Meyer, H. D.; Worth, G. A. Quantum Molecular Dynamics: Propagating Wavepackets and Density Operators Using the Multiconfiguration Time-Dependent Hartree Method. *Theor. Chem. Acc.* **2003**, *109* (5), 251–267. https://doi.org/10.1007/s00214-003-0439-1.

(33) Manthe, U. A Multilayer Multiconfigurational Time-Dependent Hartree Approach for Quantum Dynamics on General Potential Energy Surfaces. *J. Chem. Phys.* **2008**, *128* (16). https://doi.org/10.1063/1.2902982.

(34) Martínez, M. A.; Aranda, D.; Ortí, E.; Aragó, J.; Sánchez, L. Thermodynamics of the Self-Assembly of N-Annulated Perylene Bisimides in Water. Disentangling the Enthalpic and Entropic Contributions. *Org. Chem. Front.* **2023**, *10* (8), 1959–1967. https://doi.org/10.1039/d3qo00111c.

(35) Cantina, M.; Padula, D.; Segalina, A.; Giannini, S.; Santoro, F.; Prampolini, G.; Pastore, M. Anticooperative Self-Assembly of Perylene Diimide Dyes in Water Unveiled by Advanced Molecular Dynamics Simulations. *Nanoscale* **2025**, *17* (40), 23626–23641. https://doi.org/10.1039/D5NR02723C.
